# Expression and Regulatory Network Analysis of miR-140-3p, a New Potential Serum Biomarker for Autism Spectrum Disorder

**DOI:** 10.3389/fnmol.2017.00250

**Published:** 2017-08-10

**Authors:** Matilde Cirnigliaro, Cristina Barbagallo, Mariangela Gulisano, Carla N. Domini, Rita Barone, Davide Barbagallo, Marco Ragusa, Cinzia Di Pietro, Renata Rizzo, Michele Purrello

**Affiliations:** ^1^Section of Biology and Genetics Giovanni Sichel, Department of Biomedical and Biotechnological Sciences, University of Catania Catania, Italy; ^2^Section of Child and Adolescent Psychiatry, Department of Clinical and Experimental Medicine, University of Catania Catania, Italy; ^3^Associazione Oasi Maria SS. Onlus (IRCCS), Institute for Research on Mental Retardation and Brain Aging Troina, Italy

**Keywords:** ASD, miRNAs, cellular networks, CD38, NRIP1/RIP140, liquid biopsy, comorbid neurodevelopmental disorders, YGTSS

## Abstract

Given its prevalence and social impact, Autism Spectrum Disorder (ASD) is drawing much interest. Molecular basis of ASD is heterogeneous and only partially known. Many factors, including disorders comorbid with ASD, like TS (Tourette Syndrome), complicate ASD behavior-based diagnosis and make it vulnerable to bias. To further investigate ASD etiology and to identify potential biomarkers to support its precise diagnosis, we used TaqMan Low Density Array technology to profile serum miRNAs from ASD, TS, and TS+ASD patients, and unaffected controls (NCs). Through validation assays in 30 ASD, 24 TS, and 25 TS+ASD patients and 25 NCs, we demonstrated that miR-140-3p is upregulated in ASD vs.: NC, TS, and TS+ASD (Tukey's test, *p*-values = 0.03, = 0.01, < 0.0001, respectively). ΔCt values for miR-140-3p and YGTSS (Yale Global Tic Severity Scale) scores are positively correlated (Spearman *r* = 0.33; Benjamini-Hochberg *p* = 0.008) and show a linear relationship (*p* = 0.002). Network functional analysis showed that nodes controlled by miR-140-3p, especially CD38 and NRIP1 which are its validated targets, are involved in processes convergingly dysregulated in ASD, such as synaptic plasticity, immune response, and chromatin binding. Biomarker analysis proved that serum miR-140-3p can discriminate among: (1) ASD and NC (Area under the ROC curve, AUC: 0.70; sensitivity: 63.33%; specificity: 68%); (2) ASD and TS (AUC: 0.72; sensitivity: 66.66%; specificity: 70.83%); (3) ASD and TS+ASD (AUC: 0.78; sensitivity: 73.33%; specificity: 76%). Characterization of miR-140-3p network would contribute to further clarify ASD etiology. Serum miR-140-3p could represent a potential non-invasive biomarker for ASD, easy to test through liquid biopsy.

## Introduction

Autism Spectrum Disorder (ASD) is the name for a heterogeneous group of complex neurodevelopmental conditions, which are clinically defined by: (1) defects in social interaction and communication; (2) fixed interests and repetitive behaviors. Typically, ASD symptoms become fully manifest during school age and have a lifelong impact on everyday functions (American Psychiatric Association, 2013).

The broadening of the autism concept and the resulting changes in ASD categorization have increased ASD awareness and improved its diagnostic surveillance in health and educational care. This has led to an alarming rise in the number of milder cases of ASD, without co-occurring intellectual disability, in developed countries around the world.

Recently, it has been reported that ASD affects one in 68 US children and that approximately four males suffer from ASD for every female (Christensen et al., 2016). Comorbid neuropsychiatric and neurodevelopmental disorders contribute to ASD impairment, being common (70.8%) and frequently multiple (57%) in ASD children (Simonoff et al., 2008). Such conditions include social anxiety disorder, attention-deficit/hyperactivity disorder (ADHD), oppositional defiant disorder (ODD), chronic tic disorder, and obsessive-compulsive disorder (OCD).

Tourette Syndrome (TS) is a neurodevelopmental disorder characterized by considerable motor as well as behavioral impairment: it affects approximately 1% of the population with a male:female ratio of 3:1. It is clinically defined by childhood onset of multiple motor tics and at least one phonic tic, which collectively must persist for at least 12 months (American Psychiatric Association, 2013). 88% of TS patients also show comorbidity and psychopathology. Comorbidity with ADHD and OCD is very common. Co-existent psychopathologies include depression, anxiety, learning difficulties, personality disorder, ODD, and conduct disorder (Robertson, 2012). TS can cause severe difficulties in social functioning and a reduced quality of life in patients suffering from it. Characteristic, but not essential for diagnosis, symptoms include complex tics, such as echolalia and echopraxia (copying others' vocalizations and actions, respectively), palilalia and palipraxia (repeating own words/phrases and actions, respectively), coprolalia (inappropriate involuntary swearing) as well as repeating of complex words (Robertson, 2015).

It has been observed that 4.8% of ASD children also suffer from TS (Simonoff et al., 2008) and that 6–11% of TS cases show comorbidity with ASD (Robertson, 2012). TS and ASD share clinical symptomatology and many behavioral features. Genetic studies also support the existence of common susceptibility genes in both disorders (Clarke et al., 2012). The exact etiology of both disorders is still elusive.

Strong evidence suggests that ASD may arise from genetic, epigenetic and environmental factors (Abdolmaleky et al., 2015; Nardone and Elliott, 2016; Sun et al., 2016). ASD is genetically highly heterogeneous. Both inherited and de novo ASD-associated variants have been characterized in hundreds of genes. Both inherited and de novo rare genetic variants can be detected in 10–30% of ASD cases. Single common inherited variants can be found in approximately 1.1–1.2% of ASD cases; when considered cumulatively, these can explain 15–50% ASD cases. However, no common risk loci have been identified to date for ASD (Vorstman et al., 2017).

All these heterogeneous susceptibility genes for ASD converge in a small number of commonly dysregulated biological processes and pathways, like synaptic function (including long-term potentiation and calcium signaling), immune and inflammatory responses, signaling by WNT, NOTCH, SWI/SNF (*switch/sucrose non-fermentable*), and NCOR (*nuclear receptor corepressor*) complexes, and PI3K (*phosphatidylinositol-4,5-bisphosphate 3-kinase*)/mTOR (*mammalian target of rapamycin*) signaling (Levitt and Campbell, 2009; Voineagu and Eapen, 2013; Chen et al., 2014; Gokoolparsadh et al., 2016; Ansel et al., 2017).

Resultant micro- and macro-structural and functional abnormalities, which emerge during brain development in ASD, create the dysfunction of neural networks involved in socio-emotional processing (Vissers et al., 2012; Maximo et al., 2014; Kern et al., 2015; Ecker, 2017).

Since common risk loci for ASD have not been proposed yet (Vorstman et al., 2017), a molecular test for non-syndromic ASD is not available and diagnosis relies on clinical assessment and confirmation. Clinical diagnosis of ASD depends on behavioral observations, according to the Diagnostic and Statistical Manual of Mental Disorders (5th ed.; DSM–5; American Psychiatric Association, 2013). Accepted gold standard tools for diagnostic assessment of ASD are the Autism Diagnostic Observation Schedule (ADOS) and the Autism Diagnostic Interview-Revised (ADI-R) (Falkmer et al., 2013). Considering the clinical variation and etiological heterogeneity of ASD, a precise diagnosis can be very difficult. It is further complicated by ASD association with comorbid disorders (Constantino and Charman, 2016). Therefore, there is an urgent need for potential ASD biomarkers that could support clinical discrimination of patients.

MicroRNAs (miRNAs) are conserved post-transcriptional regulators of gene expression, collectively increasing the precision and robustness of gene-regulatory networks and affecting all cellular pathways, from development to metabolism (Berezikov, 2011). Other than in neoplastic and degenerative diseases, their dysregulation plays a role in several neurodevelopmental and neuropsychiatric disorders (Geaghan and Cairns, 2015).

Since their identification and characterization in serum and plasma of humans and other animals (Chen et al., 2008; Chim et al., 2008), extracellular miRNAs have attracted researchers for their potential as new non-invasive tools for diagnosis, prognosis, and treatment evaluation of many human diseases. Extracellular miRNAs can be detected in all mammalian body fluids. Stability and general consistency of levels among individuals, along with the existence of specific expression signatures in association with both physiological and pathological conditions, make circulating miRNAs appropriate biomarkers; these also suggest the prospective alternative use of liquid biopsies as sources of biomarkers in the clinic (Weiland et al., 2012; Larrea et al., 2016). Numerous studies have identified either serum or plasma circulating miRNAs as promising biomarkers for neurodevelopmental and neuropsychiatric disorders, as ADHD (Wu et al., 2015), TS (Rizzo et al., 2015), depression/anxiety disorder (Wang X. et al., 2015), posttraumatic stress disorder (Balakathiresan et al., 2014), bipolar disorder (Rong et al., 2011), schizophrenia (Wei et al., 2015), and epilepsy (Wang et al., 2015a,b; An et al., 2016), and other brain pathological conditions, like traumatic brain injury (Di Pietro et al., 2017) and vascular dementia (Ragusa et al., 2016).

Although ASD research is progressively and actively growing, only two papers have characterized circulating miRNAs in body fluids from ASD patients with a high-throughput approach (Mundalil Vasu et al., 2014; Hicks et al., 2016); none of these studies has also focused on patients affected by other neurodevelopmental disorders comorbid with autism.

We hypothesized that the serum profile of circulating miRNAs may contain some specific fingerprints for ASD which could also support in the discrimination among it and comorbid neurodevelopmental disorders. Aiming to gain more knowledge about ASD biomolecular basis and identify new potential biomarkers for this disorder, we exploited a high-throughput approach to analyze the expression of circulating miRNAs in serum from ASD, TS, and TS+ASD patients.

Following profiling of serum miRNAs through our previously published protocol (Rizzo et al., 2015; Ragusa et al., 2016), we validated serum miR-140-3p as significantly upregulated in ASD patients compared to unaffected controls (NCs), TS patients, and TS+ASD patients. Also, we demonstrated that its serum expression levels are correlated with scores from the tic scale YGTSS (Yale Global Tic Severity Scale). Then, we observed that miR-140-3p network node genes are involved in biological processes convergingly dysregulated in ASD (i.e., synaptic plasticity, immune response, and chromatin binding). Finally, through biomarker analysis, we proved that serum miR-140-3p can discriminate ASD from NC, TS, and TS+ASD.

## Materials and methods

### Patient selection

From a database of more than 2,000 patients (from the Section of Child and Adolescent Psychiatry, Department of Clinical and Experimental Medicine, University of Catania), 79 Caucasian patients, aged 3–13 years from various socio-economic contexts, were randomly recruited and studied from January to November 2016. Thirty patients affected by ASD [mean age 6.5 (standard deviation, SD 3.5); M:F 22:8], 24 patients affected by TS [mean age 8.7 (SD 5.2); M:F 21:3], and 25 patients affected by TS+ASD [(mean age 9.3 (SD 6.7); M:F 25:0] were included in the study. They were compared to 25 neurologically intact unaffected negative control (NC) individuals [mean age 9.5 (SD 3.9); M:F 16:9], recruited from local schools, without any history of either ASD or TS and who suffered from neither chronic diseases nor psychiatric disorders (Table 1).

**Table 1:** Clinical and neuropsychological features of study participants.

	**Number of participants**	**Age**	**M:F ratio**	**IQ**	**YGTSS**	**ADOS**			
						**Communication**	**Social interaction**	**Imagination**	**Repetitive and restricted behaviors**
ASD	30	6.5 (3.5)	22:8	59.4 (20.6)	0	7.3 (2.3)	7.6 (2.1)	3 (2.5)	3.5 (1.6)
TS	24	8.7 (5.2)	21:3	93.7 (19.1)	17.1 (7.9)	0	0	0	2.4 (1.6)
TS+ASD	25	9.3 (6.7)	25:0	94.6 (8.9)	22.12 (10.5)	6.4 (3.2)	8.5 (3.9)	3.1 (4.3)	3.7 (1.9)
NC	25	9.5 (3.9)	16:9	80.9 (24.5)	1.9 (1.7)	0	0	0	0

*Data are shown as means and standard deviations between parentheses. ASD, Autism Spectrum Disorder; TS, Tourette Syndrome; NC, Unaffected Control; M, male; F, female; IQ, Intelligence Quotient; YGTSS, Yale Global Tic Severity Scale; ADOS, Autism Diagnostic Observation Schedule*.

The study was approved by the local Ethics Committee. All parents gave written informed consent.

Diagnoses of ASD, TS and other clinical conditions were made according to both DSM-IV-TR (Diagnostic and Statistical Manual of Mental Disorders, IV edition–Text Revision) and DSM-5 criteria by a child neurologist (RR). All the participants were evaluated at the University Hospital Policlinico - Vittorio Emanuele of Catania. The three clinical groups (ASD, TS, and TS+ASD) and the NCs were assessed using the following scales/schedules: ADOS and ADI-R to evaluate ASD symptoms; YGTSS to evaluate presence and severity of tics. Moreover, the three clinical groups (ASD, TS, and TS+ASD) and the NCs were also assessed by a psychologist through WISC-III (Wechsler Intelligence Scale for Children, III edition) as an evaluation of both IQ (Intelligence Quotient) and cognitive functioning. Neuropsychological features of participants are summarized in Table 1.

In a previous study (Rizzo et al., 2015), we reported that only miR-429 was significantly differentially expressed (DE) in the serum of TS patients compared to NCs: TS patients were included in this experimental series aiming to compare them with the other classes of neuropsychiatric patients.

### Sample processing

Peripheral blood samples from all participants were taken in the morning using a butterfly device into serum separator collection tubes, provided with Clot activator and gel for serum separation as additives (BD Biosciences). Collection tubes were treated according to current procedures for clinical samples. In order to separate serum from blood cells, tubes were rotated end-over-end at 20°C for 30′ and then centrifuged at 3,500 rpm at 4°C for 15′ in a Beckman J2-21. Supernatants were aliquoted into 1.5 ml RNase-free tubes and stored at −80°C. Prior to RNA extraction, stored supernatants were centrifuged again at 3500 rpm at 4°C for 15′ to remove circulating cells or debris. Serum samples were aliquoted into 1.5 ml RNase-free tubes and they were either immediately used for RNA extraction or stored at −80°C until analysis (Rizzo et al., 2015).

### RNA extraction

RNA was extracted from 400 μl serum samples using Qiagen miRNeasy Mini Kit (Qiagen, GmbH, Hilden, Germany), according to Qiagen Supplementary Protocol for purification of total RNA, including small RNAs, from serum or plasma. RNA was eluted in a 40 μl total volume of RNase-free water with two consecutive steps of elution (30 μl followed by another 10 μl of RNase-free water) performed in the same collection tube.

### MiRNA profiling

We used TLDA (TaqMan Low Density Array) technology to profile the serum expression of 754 different human miRNAs of four ASD patients, five TS patients, four TS+ASD patients and three NCs. 3 μl of RNA were reverse transcribed and preamplified according to manufacturer's instructions. Preamplified products were loaded on TaqMan Human MicroRNA Array v3.0 A and B 384-well microfluidic cards (Applied Biosystems, Foster City, CA, USA). PCR reactions on TLDAs were performed on a 7900HT Fast Real Time PCR System (Applied Biosystems) (Ragusa et al., 2016).

We individually carried out the analysis on microfluidic cards A and B. We used a customized normalization approach for the relative quantification analysis. Supplementary File 1 reports Ct values for TLDA panels A and B and shows our procedure step by step. For each comparison, a Ct value matrix (miRNAs in rows, samples in columns) was created. In a similar way to the GMN (global median normalization) method (Park et al., 2003), for each sample of the comparison, the median and mean Ct values within the array, reflecting the loaded mass of template cDNA, were calculated. However, all Ct values representing a specific miRNA were kept out of these calculations if even just one of them corresponded to a flagged well. Then, using the Pearson correlation, miRNAs whose expression profile was closer (more positively correlated) to these values were identified as the best endogenous controls within the arrays. We normalized MiRNAs to the top three stable miRNAs within the arrays. MiR-146a and miR-223^*^ were the most frequently stable miRNAs for cards A and B, respectively, and the most abundant among those we could select.

Therefore, for each comparison, three ΔCt value matrices (miRNAs in rows, samples in columns) were produced according to the 2^−ΔΔCt^ method (Schmittgen and Livak, 2008). DE circulating miRNAs were obtained performing SAM (Significance of Microarrays Analysis) statistical analysis on these matrices with MeV (Multi experiment viewer v4.8.1) statistical analysis software^1^. For each pairwise comparison, we used a two-class unpaired test, based on at least 100 permutations per miRNA, with a FDR (False Discovery Rate) cut-off of 0.15, in order to detect dysregulated miRNAs. This analysis identified many DE miRNAs for each comparison. However, we have used very strict criteria to select miRNAs for further validation (i.e., number of SAM tests in which they were identified as DE, number of comparisons in which they resulted as DE, their abundance and the quality of their amplification curves during the profiling runs) in order to investigate only the most promising ones.

### MiRNA profiling data validation

RNA from sera of 30 ASD, 24 TS, and 25 TS+ASD patients and 25 NCs was used to perform miRNA-specific reverse transcription reactions producing miRNA-specific cDNAs for real-time PCRs. These RT-PCR analyses were performed using TaqMan MicroRNA Assays (Applied Biosystems) specific for the most interesting miRNAs identified as DE, miR-30d, miR-140-3p, miR-148a^*^, and miR-222, and for the selected endogenous control, miR-146a. At first, the ASD group was composed of 32 patients. We checked if some of those samples should be considered as outliers, within this original ASD group, for: (1) the serum expression of miR-140-3p; (2) the severity of ASD symptoms. We have looked at their ΔCt values for miR-140-3p and at scores that they obtained for the four items of the ADOS scale (A: Communication; B: Social interaction; C: Imagination; D: Repetitive and restricted behaviors). For these expression values and ADOS scores, we defined the corresponding mean ± 2^*^ (SD) ranges and we considered patients with a value and/or score outside of those ranges as outliers. Two ASD patients were excluded from the original ASD group since: (1) both were outliers for miR-140-3p expression; (2) one was an outlier for the Imagination item (1/4 ADOS items), whereas the other one was an outlier for the Imagination and Repetitive and restricted behaviors items (2/4 ADOS items). All the following analyses were performed with GraphPad Prism for Windows v6.01 (GraphPad Software, La Jolla California USA^2^). D'Agostino-Pearson omnibus K2 test and Shapiro-Wilk test were performed to check if data from every small group were normally distributed. Ordinary one-way ANOVA was used to test the differential expression of the selected miRNAs between the four groups. Statistical significance was established at a *p* ≤ 0.05. Tukey's multiple comparisons test was performed to identify which groups differed in the selected miRNAs' expression. Statistical significance was established at a multiplicity adjusted *p* ≤ 0.05. Expression FC (Fold Change) values of miRNAs were calculated by applying the 2^−ΔΔCt^ method (Schmittgen and Livak, 2008).

### Correlation between miR-140-3p expression and neuropsychiatric parameters

Correlation between ΔCt values for miR-140-3p, obtained from the normalization to miR-146a, and neuropsychiatric parameters was analyzed in both a general (all patients and controls) and a class-specific (just one class of patients and controls) way, since some of these parameters were related to a certain class of neuropsychiatric disorders. IQ, ADOS items regarding communication, social interaction, imagination, and repetitive and restricted behaviors (ADOS items A-D), and YGTSS (Yale Global Tic Severity Scale) were the neuropsychiatric parameters chosen for this analysis. Either Pearson or Spearman correlation was computed on GraphPad Prism software when analyzing normally and not normally distributed data, respectively. Two-sided *p*-values from this correlation analysis were corrected for multiple comparisons by using three different approaches: Bonferroni correction, Holm-Bonferroni correction, and Benjamini-Hochberg (BH) FDR procedure. Statistical significance was established at a *p*-value ≤ Bonferroni corrected α = 0.05/16 = 0.003, at a Holm-Bonferroni corrected *p* ≤ 0.05, and at a Benjamini-Hochberg FDR adjusted *p* ≤ 0.01. Linear regression analysis was also carried out on GraphPad Prism software only for significant correlations. Statistical significance was established at a *p* ≤ 0.05.

### Computational analyses

#### Reconstruction of the miR-140-3p-mediated regulatory network

MiR-140-3p targets whose validation was based on strong evidence were retrieved by DIANA-TarBase v7.0^3^ (Vlachos et al., 2015) and miRTarBase^4^ (Chou et al., 2016) databases. The biological network, composed of MIR140, these targets, and their first neighbors, was built retrieving interactome data through BisoGenet v3.0.0 Plug-in (Martin et al., 2010) in Cytoscape v3.4.0 (Shannon et al., 2003). Network centralities analysis, permitting the identification of the nodes that, more than others, were good candidates as regulators of the underlying biological processes in which the network is involved, was carried out through CentiScaPe v2.1 Plug-in (Scardoni et al., 2014).

#### Network functional analysis

clusterProfiler v3.2.11 R package (Yu et al., 2012) was used to perform functional enrichment analyses on miR-140-3p-mediated regulatory network node genes in R v3.3.2^5^ (R Core Team, 2016). We searched for the gene annotation terms from the GO (Gene Ontology), DO (Disease Ontology), KEGG (Kyoto Encyclopedia of Genes and Genomes), and Reactome databases that were over-represented in the list of network node genes compared to the entire genome. Statistical significance for the hypergeometric test was established at a BH adjusted *p* ≤ 0.05. gofilter() and simplify() functions in clusterProfiler were employed in order to select level-specific GO terms and to remove the most redundant ones, respectively.

#### Network expression analysis

In order to investigate if deregulation of network node genes was implicated in ASD, we searched for raw high-throughput gene expression datasets produced from the analysis of samples of ASD patients on two public repositories, GEO (Gene Expression Omnibus) DataSets (Edgar et al., 2002) and ArrayExpress (Kolesnikov et al., 2015). Datasets retrieved by GEO DataSets were analyzed performing limma tests with the GEO2R tool^6^ (Barrett et al., 2013), whereas datasets retrieved by ArrayExpress were analyzed performing Tusher SAM tests with MeV software. We reported only network node genes whose log_2_FC expression was significantly higher than 1 and lower than -1 as upregulated and downregulated, respectively, within ASD datasets. MeV software was also used to produce the curated ASD expression heatmap. Supplementary File 2 reports all the datasets selected for network functional analysis along with their references and IDs (Gregg et al., 2008; Kuwano et al., 2011; Voineagu et al., 2011; Ginsberg et al., 2012; Kong et al., 2012; see Supplementary File 2).

### ROC curve analysis and biomarker performance evaluation

ΔCt values for miR-140-3p, obtained from the normalization to miR-146a, served as input data to perform a classical univariate ROC (Receiver Operator Characteristic) curve analysis for each of the comparisons where we found this miRNA to be dysregulated on the server Metaboanalyst 3.0^7^ (Xia and Wishart, 2016). An appropriate ΔCt cut-off point maximizing both sensitivity and specificity (that is, the threshold that maximizes the distance to the diagonal line) was found for each curve by calculating the maximum Youden index J (max [(sensitivity + specificity)–1]). GraphPad Prism software was used to create **Figure 4**. The true positive rate (y-axis) was plotted in function of the false positive rate (x-axis), for different ΔCt cut-off points.

Since these ROC curves were based on a miRNA already identified as differentially expressed between the compared groups (miR-140-3p), through them we could only assess its idealized discriminative power. It is possible that this miRNA only accurately predicts outcomes in the initial data set and that minor fluctuations in the training data could markedly lower its predictive performance.

Therefore, after these preliminary ROC curve analyses, we built corresponding logistic regression models for the expression of miR-140-3p and we tested them through CV (cross-validation) and permutation testing, once again, by using the server Metaboanalyst 3.0. CV gives an indication of how accurate a given model might be in predicting new samples, validating its general applicability (Xia et al., 2013). 100-time repeated random sub-sampling CV was used to test the performance of the built logistic regression models. At each CV, 2/3 of samples are used for model training and the remaining 1/3 of samples are used for testing. Permutation testing indicates if a given model is significantly different from a random guessing model for the sample population, validating the proposed model structure (Xia et al., 2013). Permutation testing on the performance measure AUC (Area under the ROC curve) was used to calculate the significance of the built logistic regression models. The permutation tests use this procedure: random label re-assignment to each sample; 3-time repeated random sub-sampling CV; comparison of the performance measures between the models obtained by using the original and the permuted sample labels. This procedure was repeated 100 times. If the performance measure of the original data lies outside the normal distribution of the one of the permuted data, then the tested model is significant. Statistical significance was established at a *p* ≤ 0.05.

## Results

### High-throughput expression analysis of circulating miRNAs in ASD, TS, and TS+ASD patients

By using TLDA technology, we investigated the expression levels of 754 miRNAs in sera from four ASD patients, five TS patients, four TS+ASD patients, and three unaffected NCs. We identified miR-146a and miR-223^*^ as the best endogenous controls for panels A and B, respectively. Supplementary File 1 reports Ct values for TLDA panels A and B.

We found that four miRNAs from panel A (miR-140-3p, miR-222, miR-454, miR-483-5p), and five miRNAs from panel B (miR-30d, miR-30e-3p, miR-148a^*^, miR-1274B, miR-1290) were significantly DE in at least one of the comparisons made (FDR < 0.15 in each pairwise comparison).

### Dysregulated expression levels of miR-140-3p in serum from ASD patients

We selected miR-30d, miR-140-3p, miR-148a^*^, and miR-222 for further validation through single TaqMan assays. MiR-146a was used as endogenous control in all the analyses carried out. Supplementary File 3 reports all ΔCt values from validation assays.

**Figure 1:**
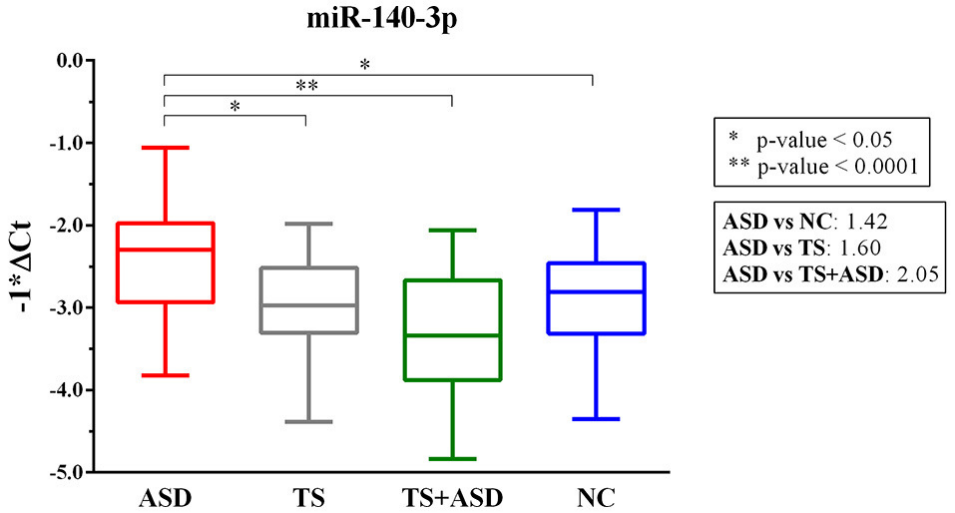
MiR-140-3p is significantly dysregulated in serum of ASD patients. Single TaqMan assay for miR-140-3p. Box and whiskers plot depicting serum levels of miR-140-3p in ASD, TS and TS+ASD patients and NCs. Y-axis represents the distribution of −1^*^ΔCt values for miR-140-3p. Multiplicity adjusted *p*-values from Tukey's multiple comparisons test and expression FC (Fold Change) values are shown in the boxes next to the plot.

We found only miR-140-3p as significantly dysregulated in serum from ASD patients (ordinary one-way ANOVA, *p* = 0.0001). In particular, serum levels of miR-140-3p were higher in 30 ASD patients compared to 25 NCs (Tukey's multiple comparisons test, multiplicity adjusted *p* = 0.03), 24 TS patients (Tukey's multiple comparisons test, multiplicity adjusted *p* = 0.01), and 25 TS+ASD patients (Tukey's multiple comparisons test, multiplicity adjusted *p* < 0.0001) (Figure 1). We did not observe any expression differences for miR-140-3p when comparing TS patients to NCs (Tukey's multiple comparisons test, multiplicity adjusted *p* = 0.98), TS+ASD patients to NCs (Tukey's multiple comparisons test, multiplicity adjusted *p* = 0.29), and TS+ASD patients to TS patients (Tukey's multiple comparisons test, multiplicity adjusted *p* = 0.53).

### Correlation between miR-140-3p expression and neuropsychiatric parameters

In order to test if any link existed between serum expression of miR-140-3p and commonly used ASD and TS neuropsychiatric parameters, we computed the correlation between ΔCt values for miR-140-3p and various neuropsychiatric scores of study participants (Table 2). Supplementary File 4 reports ΔCt value for miR-140-3p and neuropsychiatric scores of each participant.

**Table 2:** Correlation between miR-140-3p expression and neuropsychiatric parameters.

**ALL PATIENTS AND NCS**						
	**ΔCt vs. IQ**	**ΔCt vs. YGTSS**	**ΔCt vs. ADOS Communication**	**ΔCt vs. ADOS Social interaction**	**ΔCt vs. ADOS Imagination**	**ΔCt vs. ADOS Repetitive and restricted behaviors**
Spearman r	0.02	0.33	−0.13	−0.07	−0.17	NT
Pearson r	NT	NT	NT	NT	NT	−0.17
95% CI	−0.18–0.21	0.15–0.50	−0.32–0.07	−0.27–0.12	−0.36–0.03	−0.35–0.02
two-sided *p*-value	0.86	0.0005	0.18	0.45	0.09	0.08
Is *p* < Bonferroni corrected α?	N	Y	N	N	N	N
Bonferroni-Holm adjusted *p*-value	1.00	0.008	1.00	1.00	1.00	1.00
Benjamini-Hochberg *p*-value (FDR 1%)	0.86	0.008	0.29	0.51	0.23	0.23
Number of XY Pairs	104	104	104	104	104	104
**ASD PATIENTS AND NCS**						
	**ΔCt vs. ADOS Communication**	**ΔCt vs. ADOS Social interaction**	**ΔCt vs. ADOS Imagination**	**ΔCt vs. ADOS Repetitive and restricted behaviors**		
Spearman r	-0.27	-0.20	-0.31	NT		
Pearson r	NT	NT	NT	-0.29		
95% CI	−0.51–0.003	−0.45–0.08	−0.53–−0.04	−0.51–−0.02		
two-sided *p*-value	0.05	0.15	0.02	0.03		
Is *p* < Bonferroni corrected α?	N	N	N	N		
Bonferroni-Holm adjusted *p*-value	0.60	1.00	0.34	0.47		
Benjamini-Hochberg *p*-value (FDR 1%)	0.18	0.29	0.18	0.18		
Number of XY Pairs	55	55	55	55		
**TS PATIENTS AND NCS**						
	**ΔCt vs. YGTSS**					
Spearman r	−0.03					
95% CI	−0.31–0.26					
two-sided *p*-value	0.84					
Is *p* < Bonferroni corrected α?	N					
Bonferroni-Holm adjusted *p*-value	1.00					
Benjamini-Hochberg *p*-value (FDR 1%)	0.86					
Number of XY Pairs	49					
**TS**+**ASD PATIENTS AND NCS**						
	Δ**Ct vs. YGTSS**	Δ**Ct vs. ADOS Communication**	Δ**Ct vs. ADOS Social interaction**	Δ**Ct vs. ADOS Imagination**	Δ**Ct vs. ADOS Repetitive and restricted behaviors**	
Spearman r	0.21	0.17	0.15	0.17	0.20	
95% CI	−0.08–0.47	−0.12–0.43	−0.14–0.42	−0.12–0.44	−0.09–0.46	
two-sided *p*-value	0.14	0.24	0.28	0.23	0.17	
Is *p* < Bonferroni corrected α?	N	N	N	N	N	
**TS**+**ASD PATIENTS AND NCS**						
	Δ**Ct vs. YGTSS**	Δ**Ct vs. ADOS Communication**	Δ**Ct vs. ADOS Social interaction**	Δ**Ct vs. ADOS Imagination**	Δ**Ct vs. ADOS Repetitive and restricted behaviors**	
Bonferroni-Holm adjusted *p*-value	1.00	1.00	1.00	1.00	1.00	
Benjamini-Hochberg *p*-value (FDR 1%)	0.29	0.33	0.35	0.33	0.29	
Number of XY Pairs	50	50	50	50	50	

*We performed the analyses in both a general (Section 1) and a class-specific (Sections 2–4) manner. Either Pearson or Spearman r values from every analysis are reported. Bonferroni corrected α = 0.05/16 = 0.003. IQ, Intelligence Quotient; ADOS, Autism Diagnostic Observation Schedule; YGTSS, Yale Global Tic Severity Scale; 95% CI, 95% Confidence Interval; NT, Not Tested; N, no; Y, yes; FDR, False Discovery Rate*.

**Figure 2:**
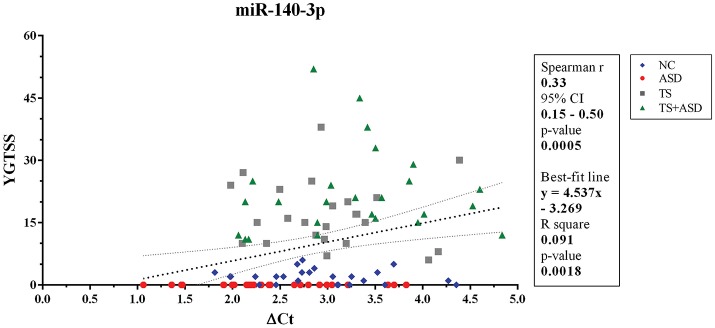
Correlation between serum levels of miR-140-3p and scores from YGTSS scale. The scatterplot refers to all the 104 analyzed samples and it also reports the best-fit line obtained from linear regression analysis. YGTSS, Yale Global Tic Severity Scale; 95% CI, 95% Confidence Interval.

When we included all patients and controls in our analysis, we found a positive correlation (Spearman *r* = 0.33; two-sided *p* = 0.0005, significant according to Bonferroni correction; Holm-Bonferroni corrected *p* = 0.008; Benjamini-Hochberg FDR adjusted *p* = 0.008) and a linear relationship (y = 4.537x–3.269, y: YGTSS score, x: ΔCt value for miR-140-3p, two-sided *p* = 0.002) between miR-140-3p expression levels and YGTSS scores (Figure 2). We could assume that lower serum levels of miR-140-3p, which have been observed in TS+ASD patients, are potentially associated with both occurrence and worsening of motor and phonic tics and that higher serum levels of miR-140-3p, which we found in ASD patients, could be linked to the absence of tics.

This analysis confirmed that serum expression of miR-140-3p correlated with a crucial neuropsychiatric scale for the clinical diagnosis of TS. We infer that miR-140-3p could prove to be useful to strengthen the behavior-based diagnosis of either ASD or TS+ASD, which can be particularly challenging in some clinical cases.

### Reconstruction of miR-140-3p-mediated regulatory network: functional and expression analyses of network node genes

By searching on online databases of miRNA-mRNA interactions for validated targets of miR-140-3p, we retrieved CD38 (*CD38 molecule*) and NRIP1 (*nuclear receptor interacting protein 1*) as its only targets whose validation was based on strong evidence. Through network analysis, we reconstructed the regulatory network composed of MIR140 (*microRNA 140*, the gene encoding miR-140-3p), CD38, NRIP1, and their first neighbors. This network had 111 nodes and 821 edges. NRIP1, POLR2A (*RNA polymerase II subunit A*), EP300 (*E1A binding protein p300*), E2F1 (*E2F transcription factor 1*), ESR1 (*estrogen receptor 1*), PHF8 (*PHD finger protein 8*), and TAF1 (*TATA-box binding protein associated factor 1*) were the nodes with the highest degree within it (Supplementary Figure 1).

In order to investigate the potential etiological role of this miRNA-mediated network in ASD, we performed functional enrichment analysis of network node genes using GO, DO, KEGG, and Reactome gene annotation databases (Supplementary Figures 2–4). Supplementary File 5 reports all the data from network functional analysis.

Genes from miR-140-3p-mediated regulatory network played a role in various mechanisms within the nervous system (i.e., neurogenesis, regulation of synaptic plasticity, long term synaptic depression, cellular response to nerve growth factor, neuron differentiation, dendrite development, and neuronal death). In addition to their role in nervous system development, they were also involved in growth regulation, endocrine system development, heart development, respiratory system development, and tongue development (Table 3, Supplementary Figure 5). MIR140 gene was annotated with the over-represented DO term physical disorder, that refers to diseases determined by a genetic abnormality, error with embryonic development, infection or compromised intrauterine environment (DOID:0080015; BH adjusted *p* = 0.027; Gene Ratio = 0.070; Background Ratio = 0.017). Among the most interesting terms whose enrichment was determined by CD38, we found those regarding response to estradiol, retinoic acid, drugs, hypoxia, ketone, and oxidative stress, activation and proliferation of immune cells, regulation of protein localization, cellular calcium ion homeostasis, and blood circulation. We found bacterial infectious disease as the only DO term among them. Finally, CD38 was directly involved in the regulation of synaptic plasticity after and in long-term synaptic depression (Table 4, Supplementary Figure 6). Among the most interesting terms whose enrichment was determined by NRIP1, there were those related to response to estradiol and steroid hormones, reproductive system development, and development of primary sexual characteristics. NRIP1 was annotated with many molecular functions (i.e., histone deacetylase binding, nuclear hormone receptor binding, core promoter sequence-specific DNA binding, retinoic acid receptor binding, and retinoid X receptor binding). Finally, NRIP1 regulated the transcription of genes involved in circadian rhythms by interacting with RORA (*RAR related orphan receptor A*) (Table 5, Supplementary Figure 7).

**Table 3:** Over-represented GO Biological Process terms regarding nervous system and development in miR-140-3p-mediated regulatory network.

**GO BP term ID**	**GO BP term description**	**BH adjusted *p*-value**	**Gene Ratio**	**Background Ratio**
GO:0061029	eyelid development in camera-type eye	2.31E-05	0.0482	0.0008
GO:1990090	cellular response to nerve growth factor stimulus	3.64E-05	0.0602	0.0021
GO:1990089	response to nerve growth factor	4.88E-05	0.0602	0.0023
GO:0048608	reproductive structure development	0.00015	0.1325	0.0244
GO:0061458	reproductive system development	0.00016	0.1325	0.0246
GO:0016049	cell growth	0.00048	0.1325	0.0289
GO:0048732	gland development	0.00061	0.1205	0.0244
GO:0001654	eye development	0.00066	0.1084	0.0197
GO:0051961	negative regulation of nervous system development	0.00068	0.0964	0.0152
GO:0048714	positive regulation of oligodendrocyte differentiation	0.00089	0.0361	0.0009
GO:0008584	male gonad development	0.00089	0.0723	0.0080
GO:0046546	development of primary male sexual characteristics	0.00089	0.0723	0.0080
GO:0008406	gonad development	0.0012	0.0843	0.0125
GO:0030850	prostate gland development	0.0012	0.0482	0.0028
GO:0070997	neuron death	0.0012	0.0964	0.0169
GO:0043010	camera-type eye development	0.0013	0.0964	0.0171
GO:0045137	development of primary sexual characteristics	0.0013	0.0843	0.0128
GO:0001558	regulation of cell growth	0.0016	0.1084	0.0229
GO:0061448	connective tissue development	0.0020	0.0843	0.0139
GO:0030308	negative regulation of cell growth	0.0020	0.0723	0.0098
GO:0007507	heart development	0.0021	0.1205	0.0296
GO:0050768	negative regulation of neurogenesis	0.0021	0.0843	0.0141
GO:1901215	negative regulation of neuron death	0.0024	0.0723	0.0102
GO:2000171	negative regulation of dendrite development	0.0028	0.0361	0.0015
GO:0045665	negative regulation of neuron differentiation	0.0033	0.0723	0.0111
GO:0061196	fungiform papilla development	0.0035	0.0241	0.0004
GO:0001893	maternal placenta development	0.0037	0.0361	0.0017
GO:0048713	regulation of oligodendrocyte differentiation	0.0037	0.0361	0.0017
GO:0010721	negative regulation of cell development	0.0037	0.0843	0.0163
GO:0060541	respiratory system development	0.0041	0.0723	0.0118
GO:0048709	oligodendrocyte differentiation	0.0053	0.0482	0.0047
GO:0035265	organ growth	0.0054	0.0602	0.0084
GO:0060534	trachea cartilage development	0.0054	0.0241	0.0005
GO:0007423	sensory organ development	0.0063	0.1084	0.0298
GO:0045926	negative regulation of growth	0.0069	0.0723	0.0136
GO:0060433	bronchus development	0.0088	0.0241	0.0007
GO:0060525	prostate glandular acinus development	0.0088	0.0241	0.0007
GO:1901214	regulation of neuron death	0.0098	0.0723	0.0150
GO:0060736	prostate gland growth	0.0098	0.0241	0.0007
GO:0060742	epithelial cell differentiation involved in prostate gland development	0.0098	0.0241	0.0007
GO:0030323	respiratory tube development	0.011	0.0602	0.0104
GO:0060348	bone development	0.011	0.0602	0.0105
GO:0051216	cartilage development	0.012	0.0602	0.0108
GO:0003417	growth plate cartilage development	0.012	0.0241	0.0008
GO:0001501	skeletal system development	0.015	0.0964	0.0290
GO:0035855	megakaryocyte development	0.015	0.0241	0.0010
GO:0071696	ectodermal placode development	0.015	0.0241	0.0010
GO:0048638	regulation of developmental growth	0.016	0.0723	0.0173
GO:0010977	negative regulation of neuron projection development	0.016	0.0482	0.0073
GO:0060749	mammary gland alveolus development	0.017	0.0241	0.0011
GO:0061377	mammary gland lobule development	0.017	0.0241	0.0011
GO:0071560	cellular response to transforming growth factor beta stimulus	0.018	0.0602	0.0124
GO:0035270	endocrine system development	0.018	0.0482	0.0077
GO:0071559	response to transforming growth factor beta	0.018	0.0602	0.0125
GO:0060438	trachea development	0.018	0.0241	0.0011
GO:0061180	mammary gland epithelium development	0.018	0.0361	0.0038
GO:0003416	endochondral bone growth	0.020	0.0241	0.0012
GO:0043586	tongue development	0.020	0.0241	0.0012
GO:0048167	regulation of synaptic plasticity	0.021	0.0482	0.0081
GO:0030325	adrenal gland development	0.021	0.0241	0.0013
GO:0001890	placenta development	0.022	0.0482	0.0083
GO:0035264	multicellular organism growth	0.022	0.0482	0.0083
GO:0060351	cartilage development involved in endochondral bone morphogenesis	0.023	0.0241	0.0013
GO:0098868	bone growth	0.023	0.0241	0.0013
GO:0060292	long term synaptic depression	0.024	0.0241	0.0014
GO:0010720	positive regulation of cell development	0.025	0.0843	0.0262
GO:0007176	regulation of epidermal growth factor-activated receptor activity	0.027	0.0241	0.0015
GO:0030878	thyroid gland development	0.027	0.0241	0.0015
GO:0060537	muscle tissue development	0.029	0.0723	0.0206
GO:0030900	forebrain development	0.030	0.0723	0.0209
GO:0048736	appendage development	0.036	0.0482	0.0101
GO:0060173	limb development	0.036	0.0482	0.0101
GO:0030324	lung development	0.037	0.0482	0.0102
GO:0008585	female gonad development	0.037	0.0361	0.0055
GO:0050769	positive regulation of neurogenesis	0.038	0.0723	0.0225
GO:0045684	positive regulation of epidermis development	0.038	0.0241	0.0019
GO:0060612	adipose tissue development	0.038	0.0241	0.0019
GO:0098751	bone cell development	0.038	0.0241	0.0019
GO:0046545	development of primary female sexual characteristics	0.040	0.0361	0.0057
GO:0048738	cardiac muscle tissue development	0.042	0.0482	0.0108
GO:0021761	limbic system development	0.042	0.0361	0.0058
GO:0031076	embryonic camera-type eye development	0.043	0.0241	0.0021
GO:0016358	dendrite development	0.047	0.0482	0.0113

*Term database ID, term description, corresponding BH adjusted p-value generated by the hypergeometric test, Gene Ratio, and Background Ratio values are reported for all the GO Biological Process terms regarding nervous system and development. Gene Ratio: the ratio of number of genes of interest that are annotated with a certain term from the database used to perform the analysis to number of genes of interest that are annotated with terms from the same database. Background Ratio: the ratio of number of genes in the genome that are annotated with a certain term from the database used to perform the analysis to number of genome genes that are annotated with terms from the same database. GO, Gene Ontology; BP, Biological Process; BH, Benjamini-Hochberg*.

**Table 4:** Over-represented GO, DO and KEGG terms associated with CD38 in miR-140-3p-mediated regulatory network.

**Annotation Database**	**Term ID**	**Term description**	**BH adjusted *p*-value**	**Gene Ratio**	**Background Ratio**
GO BP	GO:0048545	response to steroid hormone	2.94E-35	0.4337	0.0221
GO BP	GO:0032355	response to estradiol	5.13E-08	0.1205	0.0070
GO BP	GO:0070482	response to oxygen levels	4.53E-07	0.1566	0.0181
KEGG	hsa05169	Epstein-Barr virus infection	3.36E-06	0.1884	0.0283
GO BP	GO:0001101	response to acid chemical	7.61E-06	0.1325	0.0158
GO BP	GO:0042493	response to drug	7.61E-06	0.1566	0.0238
GO BP	GO:0002764	immune response-regulating signaling pathway	3.86E-05	0.1566	0.0294
GO BP	GO:0051251	positive regulation of lymphocyte activation	4.72E-05	0.1205	0.0166
GO BP	GO:0036293	response to decreased oxygen levels	5.76E-05	0.1205	0.0171
GO BP	GO:0002768	immune response-regulating cell surface receptor signaling pathway	6.41E-05	0.1325	0.0217
GO BP	GO:0002694	regulation of leukocyte activation	7.86E-05	0.1446	0.0269
GO BP	GO:0002696	positive regulation of leukocyte activation	8.41E-05	0.1205	0.0180
GO BP	GO:0002757	immune response-activating signal transduction	8.67E-05	0.1446	0.0274
GO BP	GO:0050867	positive regulation of cell activation	9.75E-05	0.1205	0.0185
GO BP	GO:0051249	regulation of lymphocyte activation	0.00012	0.1325	0.0238
GO BP	GO:0050865	regulation of cell activation	0.00013	0.1446	0.0289
GO BP	GO:0002429	immune response-activating cell surface receptor signaling pathway	0.00015	0.1205	0.0197
GO BP	GO:0001666	response to hypoxia	0.00025	0.1084	0.0166
GO BP	GO:0032526	response to retinoic acid	0.00028	0.0723	0.0061
GO BP	GO:0016049	cell growth	0.00048	0.1325	0.0289
GO BP	GO:0007565	female pregnancy	0.00055	0.0843	0.0107
GO BP	GO:0044706	multi-multicellular organism process	0.0010	0.0843	0.0121
GO BP	GO:0050708	regulation of protein secretion	0.0015	0.1084	0.0226
GO BP	GO:0001558	regulation of cell growth	0.0016	0.1084	0.0229
GO BP	GO:0050851	antigen receptor-mediated signaling pathway	0.0017	0.0843	0.0134
GO BP	GO:0032844	regulation of homeostatic process	0.0032	0.1084	0.0260
GO BP	GO:0050670	regulation of lymphocyte proliferation	0.0033	0.0723	0.0111
GO BP	GO:0032944	regulation of mononuclear cell proliferation	0.0034	0.0723	0.0112
GO BP	GO:0070663	regulation of leukocyte proliferation	0.0039	0.0723	0.0117
GO BP	GO:0009306	protein secretion	0.0042	0.1084	0.0276
GO BP	GO:1904951	positive regulation of establishment of protein localization	0.0063	0.1084	0.0299
GO BP	GO:0046651	lymphocyte proliferation	0.0094	0.0723	0.0148
GO BP	GO:0032943	mononuclear cell proliferation	0.0097	0.0723	0.0149
GO BP	GO:0050796	regulation of insulin secretion	0.0098	0.0602	0.0101
GO BP	GO:0070661	leukocyte proliferation	0.012	0.0723	0.0158
GO BP	GO:0010817	regulation of hormone levels	0.012	0.0964	0.0276
GO BP	GO:0006979	response to oxidative stress	0.014	0.0843	0.0226
GO BP	GO:0090276	regulation of peptide hormone secretion	0.015	0.0602	0.0116
GO BP	GO:0030073	insulin secretion	0.015	0.0602	0.0118
GO BP	GO:0044057	regulation of system process	0.016	0.0964	0.0295
GO BP	GO:0002791	regulation of peptide secretion	0.016	0.0602	0.0119
GO BP	GO:0090087	regulation of peptide transport	0.016	0.0602	0.0121
GO BP	GO:0050671	positive regulation of lymphocyte proliferation	0.017	0.0482	0.0074
GO BP	GO:0032946	positive regulation of mononuclear cell proliferation	0.017	0.0482	0.0075
GO BP	GO:0050853	B cell receptor signaling pathway	0.018	0.0361	0.0038
GO BP	GO:0070665	positive regulation of leukocyte proliferation	0.019	0.0482	0.0079
GO BP	GO:0048167	regulation of synaptic plasticity	0.021	0.0482	0.0081
DO	DOID:104	bacterial infectious disease	0.022	0.1047	0.0338
GO BP	GO:0060292	long term synaptic depression	0.024	0.0241	0.0014
GO BP	GO:0030072	peptide hormone secretion	0.026	0.0602	0.0140
GO BP	GO:0042113	B cell activation	0.027	0.0602	0.0142
DO	DOID:0050338	primary bacterial infectious disease	0.027	0.0930	0.0297
GO BP	GO:0002790	peptide secretion	0.028	0.0602	0.0145
GO BP	GO:0046883	regulation of hormone secretion	0.030	0.0602	0.0148
GO BP	GO:1901654	response to ketone	0.032	0.0482	0.0097
GO BP	GO:0015833	peptide transport	0.033	0.0602	0.0154
GO BP	GO:0006874	cellular calcium ion homeostasis	0.037	0.0723	0.0222
GO BP	GO:0008015	blood circulation	0.037	0.0843	0.0290
GO BP	GO:0003013	circulatory system process	0.038	0.0843	0.0293
GO BP	GO:0055074	calcium ion homeostasis	0.041	0.0723	0.0230
GO BP	GO:0042886	amide transport	0.041	0.0602	0.0166
GO BP	GO:0072503	cellular divalent inorganic cation homeostasis	0.046	0.0723	0.0237
GO BP	GO:0046879	hormone secretion	0.047	0.0602	0.0173

*Term database, term ID, term description, corresponding BH adjusted p-value generated by the hypergeometric test, Gene Ratio, and Background Ratio values are reported for all the over-represented terms with which CD38 is annotated. For an in-depth description of the column names see Table 3 legend. GO, Gene Ontology; BP, Biological Process; DO, Disease Ontology; KEGG, Kyoto Encyclopedia of Genes and Genomes; BH, Benjamini-Hochberg*.

**Table 5:** Over-represented GO and Reactome terms associated with NRIP1 in miR-140-3p-mediated regulatory network.

**Annotation database**	**Term ID**	**Term description**	**BH adjusted *p*-value**	**Gene Ratio**	**Background ratio**
GO BP	GO:0043401	steroid hormone mediated signaling pathway	8.14E-41	0.398	0.011
GO BP	GO:0071383	cellular response to steroid hormone stimulus	1.44E-40	0.422	0.014
GO BP	GO:0030522	intracellular receptor signaling pathway	1.44E-40	0.434	0.016
GO BP	GO:0009755	hormone-mediated signaling pathway	1.45E-38	0.398	0.013
GO BP	GO:0048545	response to steroid hormone	2.94E-35	0.434	0.022
GO BP	GO:0071396	cellular response to lipid	9.41E-34	0.458	0.029
GO BP	GO:0071407	cellular response to organic cyclic compound	1.55E-33	0.458	0.030
GO MF	GO:0008134	transcription factor binding	4.77E-24	0.373	0.031
GO MF	GO:0003713	transcription coactivator activity	1.56E-14	0.229	0.018
GO MF	GO:0035257	nuclear hormone receptor binding	3.23E-12	0.157	0.008
GO BP	GO:0030518	intracellular steroid hormone receptor signaling pathway	9.27E-12	0.157	0.007
GO MF	GO:0051427	hormone receptor binding	2.09E-11	0.157	0.009
GO CC	GO:0000790	nuclear chromatin	1.09E-10	0.190	0.017
GO CC	GO:0044454	nuclear chromosome part	1.16E-10	0.226	0.028
GO CC	GO:0000785	chromatin	1.61E-10	0.214	0.025
GO MF	GO:0001047	core promoter binding	6.28E-10	0.145	0.010
GO MF	GO:0035258	steroid hormone receptor binding	4.19E-09	0.108	0.005
GO BP	GO:0048511	rhythmic process	6.87E-09	0.181	0.019
GO BP	GO:0007623	circadian rhythm	3.12E-08	0.145	0.011
GO BP	GO:0032355	response to estradiol	5.13E-08	0.120	0.007
GO MF	GO:0042826	histone deacetylase binding	5.96E-08	0.108	0.006
GO MF	GO:0001046	core promoter sequence-specific DNA binding	7.31E-07	0.096	0.006
GO MF	GO:0042974	retinoic acid receptor binding	1.63E-06	0.060	0.001
GO MF	GO:0003714	transcription corepressor activity	2.20E-06	0.120	0.013
GO MF	GO:0046965	retinoid X receptor binding	8.63E-06	0.048	0.001
GO BP	GO:0032922	circadian regulation of gene expression	2.31E-05	0.072	0.003
GO BP	GO:0030521	androgen receptor signaling pathway	3.24E-05	0.072	0.004
GO BP	GO:0048608	reproductive structure development	0.00015	0.133	0.024
GO MF	GO:0035259	glucocorticoid receptor binding	0.00016	0.036	0.001
GO BP	GO:0061458	reproductive system development	0.00016	0.133	0.025
GO CC	GO:0000118	histone deacetylase complex	0.00019	0.060	0.003
GO MF	GO:0030331	estrogen receptor binding	0.00024	0.048	0.002
GO MF	GO:0050681	androgen receptor binding	0.00033	0.048	0.002
GO BP	GO:0019915	lipid storage	0.00035	0.060	0.004
GO BP	GO:0051235	maintenance of location	0.00035	0.108	0.018
GO BP	GO:0007548	sex differentiation	0.00084	0.096	0.016
Reactome	400253	Circadian Clock	0.00091	0.069	0.005
GO BP	GO:0008406	gonad development	0.0012	0.084	0.012
GO BP	GO:0045137	development of primary sexual characteristics	0.0013	0.084	0.013
Reactome	1368082	RORA activates gene expression	0.0018	0.042	0.001
DO	DOID:3308	embryonal carcinoma	0.0028	0.047	0.003
GO BP	GO:0071392	cellular response to estradiol stimulus	0.0040	0.036	0.002
GO BP	GO:0010876	lipid localization	0.011	0.084	0.021
DO	DOID:11612	polycystic ovary syndrome	0.020	0.081	0.021
DO	DOID:688	embryonal cancer	0.020	0.128	0.047
DO	DOID:2994	germ cell cancer	0.029	0.128	0.051
GO BP	GO:0022602	ovulation cycle process	0.030	0.036	0.005
GO BP	GO:0008585	female gonad development	0.037	0.036	0.005
GO BP	GO:0046545	development of primary female sexual characteristics	0.040	0.036	0.006
GO BP	GO:0042698	ovulation cycle	0.048	0.036	0.006

*Term database, term ID, term description, corresponding BH adjusted p-value generated by the hypergeometric test, Gene Ratio, and Background Ratio values are reported for all the over-represented terms with which NRIP1 is annotated. For an in-depth description of the column names see Table 3 legend. GO, Gene Ontology; BP, Biological Process; MF, Molecular Function; CC, Cellular Component; DO, Disease Ontology; BH, Benjamini-Hochberg*.

**Figure 3:**
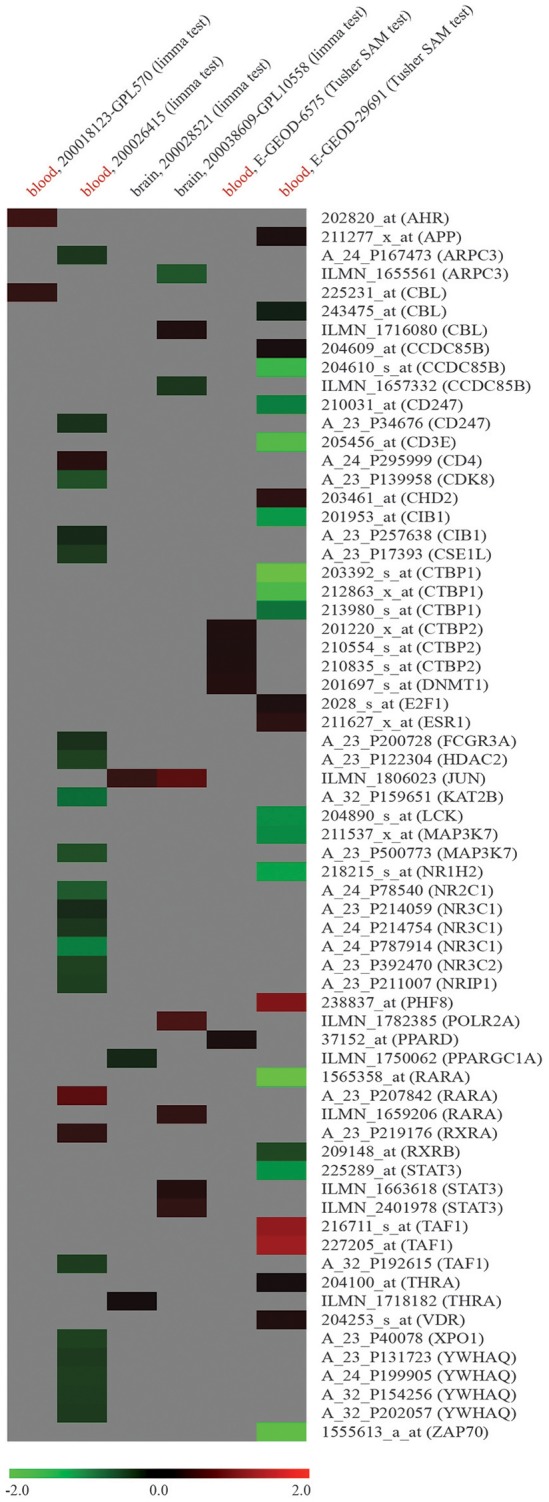
Expression analysis of node genes within miR-140-3p-mediated regulatory network in human ASD high-throughput gene expression datasets retrieved from GEO DataSets and ArrayExpress. Datasets used for the expression analysis (human ASD source tissue, dataset ID, platform type, statistical test performed) and microarray probe IDs along with their corresponding gene symbols are reported in columns and lines of this gene expression heatmap, respectively. Colored heatmap cells represent genes that are DE in a certain dataset. Data are shown as log_2_FC expression values. For more information about gene expression trend, see figure legend.

To verify if dysregulation of network node genes was implicated in ASD, we used publicly available raw high-throughput gene expression datasets produced from the analysis of ASD samples. Ten genes were found to be downregulated in whole blood of ASD patients compared to NCs (log_2_FC < -1): CCDC85B (*coiled-coil domain containing 85B*), CD3E (*CD3e molecule*), CIB1 (*calcium and integrin binding 1*), CTBP1 (*C-terminal binding protein 1*), LCK (*LCK proto-oncogene, Src family tyrosine kinase*), MAP3K7 (*mitogen-activated protein kinase kinase kinase 7*), NR1H2 (*nuclear receptor subfamily 1 group H member 2*), RARA (*retinoic acid receptor alpha*), STAT3 (*signal transducer and activator of transcription 3*), and ZAP70 (*zeta chain of T cell receptor associated protein kinase 70*); two were found to be overexpressed (log_2_FC > 1): PHF8 and TAF1 (Figure 3). Supplementary File 2 reports all the data from network expression analysis.

### Serum levels of miR-140-3p in the discrimination of ASD patients

**Figure 4:**
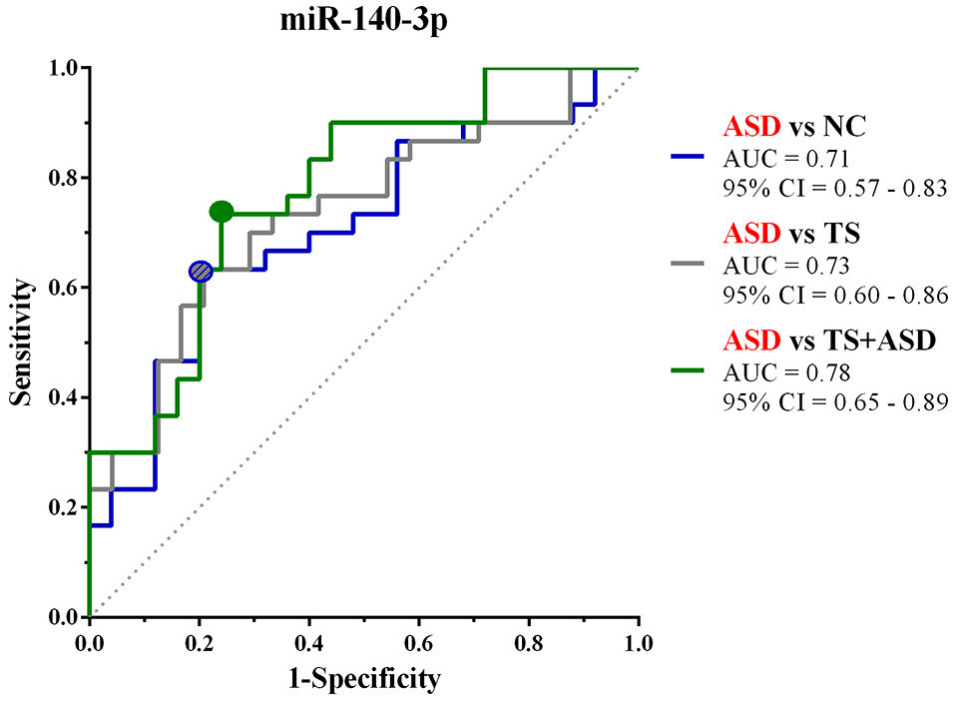
Classical univariate ROC curve analyses for the comparisons in which miR-140-3p is dysregulated. This graph compares three ROC curves, one for each comparison where we found miR-140-3p to be dysregulated. Each point on the ROC curves represents a sensitivity/specificity pair corresponding to a particular decision threshold (ΔCt value cut-off). Circles on the curves refer to the sensitivity/specificity pairs with the highest Youden index J. AUC, Area under the ROC curve; 95% CI, 95% Confidence Interval.

**Figure 5:**
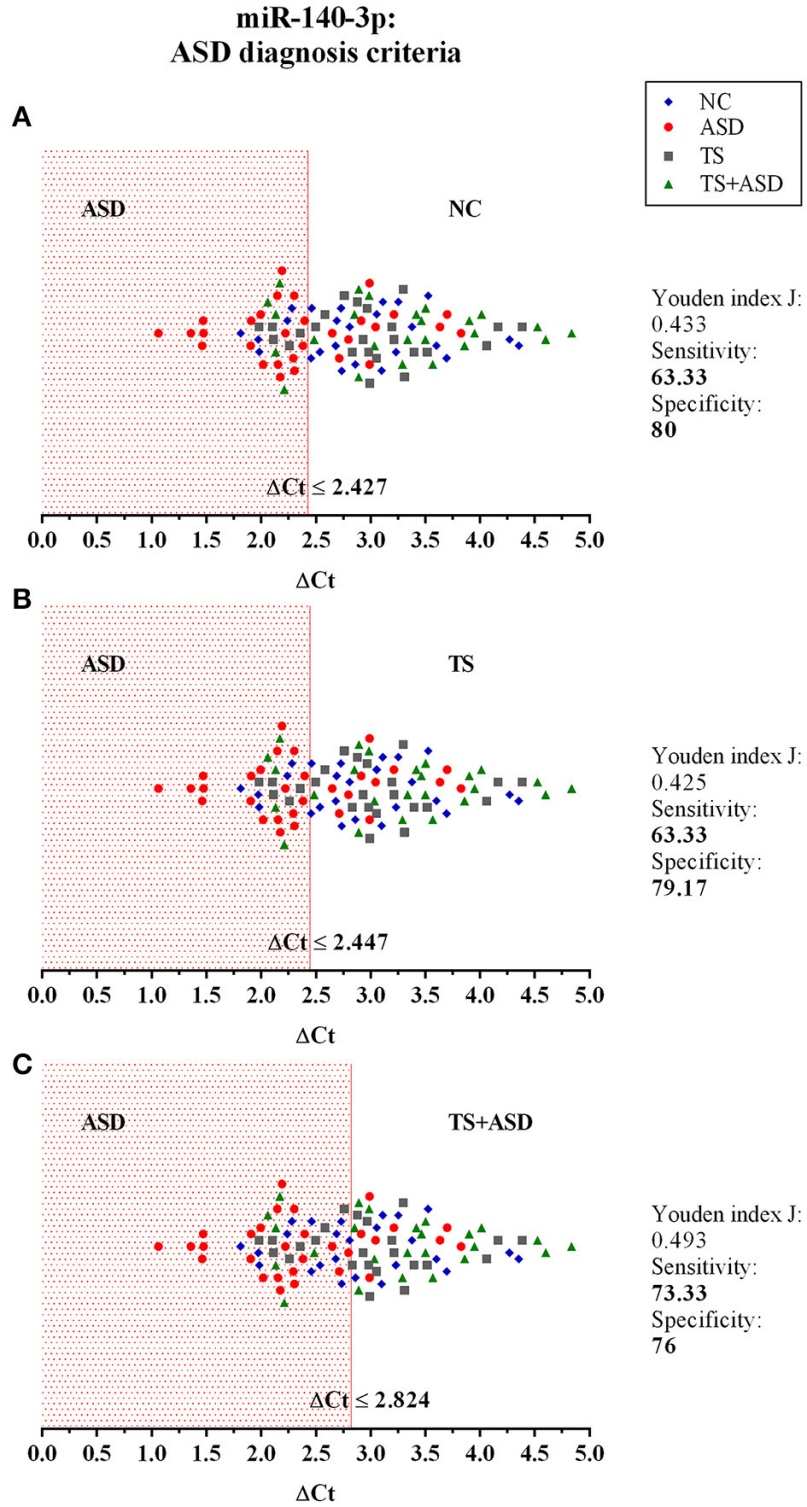
The potential use of serum miR-140-3p as a biomarker: criteria for ASD diagnosis. The graphs show the distribution of ΔCt values of all the 104 analyzed samples, for which we already had a clinical diagnosis. We used data from classical univariate ROC curve analyses to perform a blind diagnosis of all study participants. In **(A)**, the ΔCt ≤ 2.427 criterion divides ASD patients from NCs and determines the correct discrimination of 19/32 ASD patients and 20/25 NCs. In **(B)**, the ΔCt ≤ 2.447 criterion divides ASD patients from TS patients and determines the correct discrimination of 19/32 ASD patients and 19/24 TS patients. In **(C)**, the ΔCt ≤ 2.824 criterion separates ASD patients from TS+ASD patients and determines the correct discrimination of 22/32 ASD patients and 19/25 TS+ASD patients.

We used ΔCt values for miR-140-3p to perform a classical univariate ROC curve analysis for each of the comparisons where we found this miRNA to be dysregulated. The univariate ROC plots revealed an AUC of 0.71 for the comparison ASD vs. NC (*p* = 0.006), 0.73 for ASD vs. TS (*p* = 0.002), and 0.78 for ASD vs. TS+ASD (*p* = 0.00007) (Figure 4). We used ΔCt value cut-offs corresponding to the sensitivity/specificity pair with the highest Youden index J for every computed ROC curve to perform a blind diagnosis on all the 104 analyzed samples (Figure 5).

**Figure 6:**
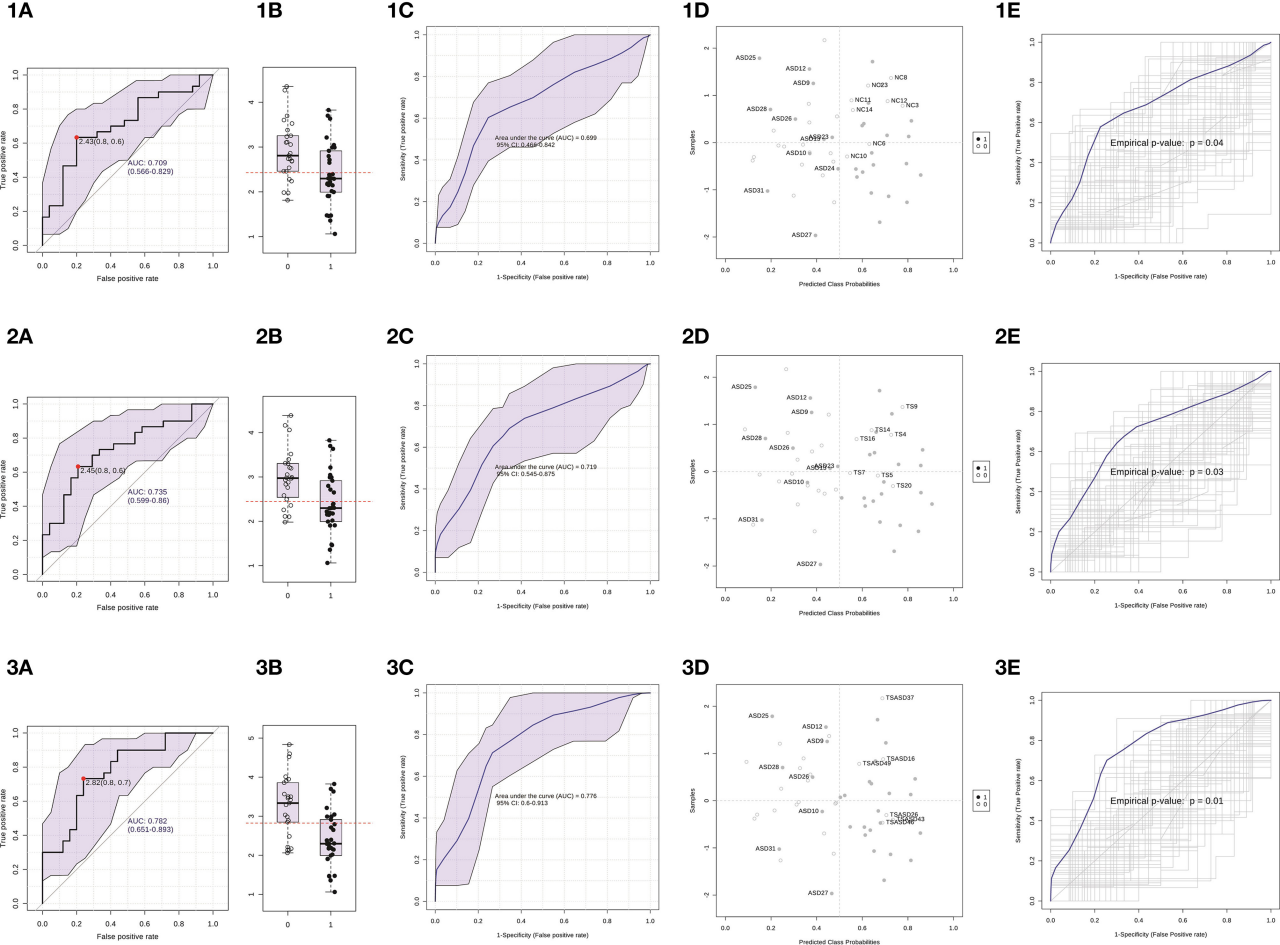
Serum miR-140-3p could be used to discriminate ASD patients. (1) The graphs refer to the comparison ASD vs. NC. (2) The graphs refer to the comparison ASD vs. TS. (3) The graphs refer to the comparison ASD vs. TS+ASD. **(A)** Classical univariate ROC curve analysis. The red dot represents the sensitivity/specificity pair with the highest Youden index J. **(B)** Boxplot depicting the distribution of ΔCt values in the two groups. The red line represents the ΔCt value cut-off corresponding to the red dot on the curve in **(A)**. The label 1 refers to the ASD group, 0 to the other group. **(C)** Average ROC curve from 100-time repeated random sub-sampling CV of the built logistic regression model. **(D)** Average predicted class probabilities (x-axis) of each sample (y-axis) from the 100 CV iterations. Probability scores more than 0.5 belong to the ASD group, those less than 0.5 belong to the other group. Incorrectly classified subjects are identified by their ID number. **(E)** Results from the permutation tests on the model performance measure AUC. Average ROC curve and corresponding *p*-value are reported. AUC, Area under the ROC curve; 95% CI: 95% Confidence Interval; CV, cross-validation.

Then, we built a logistic regression model for miR-140-3p expression in each comparison and we tested those predictive models through CV and permutation testing. 100-time repeated random sub-sampling CV was used to test the performance of the logistic regression models. MiR-140-3p continued to perform at a good level for the comparison ASD vs. NC, with an average AUC of 0.70, a sensitivity of 63.33%, and a specificity of 68% (Figures 6 1C,1D). MiR-140-3p continued to perform at a good level also for the comparison ASD vs. TS, with an average AUC of 0.72, a sensitivity of 66.66%, and a specificity of 70.83% (Figures 6 2C,2D). MiR-140-3p continued to perform at a very high level for the comparison ASD vs. TS+ASD, with an average AUC of 0.78, a sensitivity of 73.33%, and a specificity of 76% (Figures 6 3C,3D). CV results demonstrated the general applicability of these predictive models. 100-time repeated permutation tests on the performance measure AUC were carried out to validate the structure of these models. Permutation testing results were significant and quite stable in different runs for all the models tested (Figures 6 1E, 2E, and 3E).

These data proved that serum miR-140-3p could be used in the discrimination of ASD patients. In particular, it could potentially support the differential behavior-based diagnostic process of two classes of neurodevelopmental disorders, ASD and TS+ASD.

## Discussion

MiRNAs are conserved post-transcriptional regulators of gene expression, coordinating development, homeostasis, and response to external factors in organisms (Berezikov, 2011). MiRNAs are main players also in the brain, where they control many neurodevelopmental processes, including patterning, cell specification, local translational control of neuronal plasticity, neurogenesis, and neuronal apoptosis (Kosik, 2006). Some miRNAs show differential spatio-temporal and sex-biased expression patterns in the developing human brain and regulate targets that are highly enriched for genes related to transcriptional regulation, neurodevelopmental processes, and common neurodevelopmental disorders, such as ASD, schizophrenia, and bipolar disorder (Ziats and Rennert, 2014). Since miRNAs are critical for various developmental mechanisms and pathologic transcriptional processes, many studies have investigated their role in neurodevelopmental and psychiatric disorders (Omran et al., 2012; Geaghan and Cairns, 2015; Scott et al., 2015), providing some clues to the etiology of these complex disorders.

ASDs are a group of heterogeneous neurodevelopmental conditions whose exact etiology still remains unknown. Altered expression patterns of miRNAs have been found in many tissues and cells from ASD patients, such as brain cortex regions and cerebellum (Ander et al., 2015; Mor et al., 2015; Wu et al., 2016; Schumann et al., 2017), olfactory mucosal stem cells (Nguyen et al., 2016), peripheral whole blood (Huang et al., 2015a,b), and lymphoblastoid cell lines (LBC) (Sarachana et al., 2010; Ghahramani Seno et al., 2011).

Many factors, including disorders comorbid with ASD, like TS, complicate ASD behavior-based diagnosis and make it vulnerable to bias. Stability, general consistency of expression among individuals, and condition-specific expression profile make circulating miRNAs appropriate non-invasive diagnostic biomarkers (Weiland et al., 2012; Larrea et al., 2016). Expression profiling of miRNAs circulating in either serum or plasma has already been used as a biomarker discovery approach for several psychiatric disorders (Rong et al., 2011; Balakathiresan et al., 2014; Rizzo et al., 2015; Wang et al., 2015a,b; Wang X. et al., 2015; Wei et al., 2015; Wu et al., 2015; An et al., 2016; Ragusa et al., 2016; Di Pietro et al., 2017).

We hypothesized that serum profile of circulating miRNAs may contain specific fingerprints for ASD: these could provide some hints to the molecular basis of ASD and also be used as supportive means to the clinical diagnostic process, especially in the discrimination among neurodevelopmental disorders.

Using TLDA technology, we profiled serum expression of 754 human miRNAs in a discovery set of samples, including four ASD, five TS, and four TS+ASD patients and three NCs. The undeniable limit of this profiling approach is that, despite analyzing a total number of 16 samples in this exploratory step, we could have missed some significantly dysregulated circulating miRNAs because of the small number of samples in each compared group. However, we also applied very strict selection criteria for the identification of those 9 miRNAs (miR-30d, miR-30e-3p, miR-140-3p, miR-148a^*^, miR-222, miR-454, miR-483-5p, miR-1274B, and miR-1290) as differentially expressed, in order to spot more robust findings, likely to be confirmed in the next validation step. Nevertheless, through single TaqMan assays we observed the dysregulation of just one (miR-140-3p) of the 4 miRNAs selected for validation (miR-30d, miR-140-3p, miR-148a^*^, and miR-222) even if, according to results from profiling, those showed the most interesting and marked expression trends. This could be explained by the fact that TLDA approach is still a high-throughput approach: the preliminary step of preamplification suggested for TLDA analysis may have inserted an amplification bias, leading to not very accurate results. Knowing how crucial validation is, we have also performed this step by TaqMan assay, a probe-based system that is designed to specifically detect the expression of miRNAs of interests.

There is no consensus about optimal normalization strategies and accurate reference genes for both intracellular and extracellular miRNAs (Schwarzenbach et al., 2015). Suggested endogenous controls for miRNAs differ depending on: (1) the species considered; (2) either the tissue or body fluid analyzed; (3) either the physiological or pathological condition investigated; (4) different sample preparation methods, especially for circulating miRNAs (Marabita et al., 2016). On one hand, researchers can select reference miRNA genes according to reports from similar studies in the literature, and then, validate them for the set of samples under analysis (Schwarzenbach et al., 2015). On the other hand, they can screen the specific samples for more suitable endogenous controls through the profiling of a large number of genes. In this case, it is suggested to use either the mean expression values of whole miRNAs in each of the samples or genes with expression levels similar to these values as the screened endogenous controls (Schwarzenbach et al., 2015; Marabita et al., 2016). At the moment, accurately described normalization approaches and validated reference genes for serum miRNAs in ASD patients lack: this, together with different ethnicity of participants and other potential diverse analytic variables in studies similar to ours, led us to prefer our customized normalization approach over reference gene selection from literature. This approach, which is described in Supplementary File 1 and is inspired to the recommended miRNA array normalization strategy reported above (Schwarzenbach et al., 2015; Marabita et al., 2016), has proved its value in other works on neurological disorders published by our group (Rizzo et al., 2015; Ragusa et al., 2016). Thanks to it, we have selected miR-146a and miR-223^*^ as the most appropriate and accurate reference genes for our system. Some studies have identified miR-146a as either dysregulated in human ASD tissues (Mor et al., 2015; Nguyen et al., 2016) or associated to inflammation and immune response observed in neurodegenerative and neurological disorders (Iyer et al., 2012; Kiko et al., 2014; Müller et al., 2014; Wang et al., 2015b; An et al., 2016; Romano et al., 2017): however, this neither discourages its use as reference miRNA in our dataset nor affects the applicability of our results from differential expression analysis.

Through our expression analysis, we have identified miR-140-3p as dysregulated in serum from ASD patients. It is upregulated in ASD patients compared to NCs, TS patients, and TS+ASD patients: its levels are the highest in the ASD group (Figure 1). We observed that miR-140-3p levels are the lowest in the TS+ASD group. It is interesting that the two groups ASD and TS+ASD showed such a different expression trend for this miRNA. It is likely that the lower miR-140-3p expression reflects the presence of phonic and motor tics due to the comorbidity of TS with ASD. It may also depend on other either physiological conditions or comorbidities: this needs to be further investigated. On the contrary, according to our previous study on TS (Rizzo et al., 2015), we expected not to find any difference in miR-140-3p expression between TS patients and NCs.

According to expression data from the Human miRNA Tissue Atlas (Ludwig et al., 2016), miR-140-3p is highly expressed in bone, nerves, arteries, meninges (arachnoid mater and dura mater), muscle, and adipose tissue. Published data also confirm that miR-140-3p is one of the top highly expressed miRNAs in the human brain cortex (Shao et al., 2010) and that it is not specific for any of the different human blood cell compounds (Leidinger et al., 2014). The intracellular roles exerted by miR-140-3p have been mainly investigated in human pathologies, such as cancer (Lionetti et al., 2009; Tan et al., 2011; Miles et al., 2012; Piepoli et al., 2012; Sand et al., 2012a,b; Serrano et al., 2012; Bayrak et al., 2013; Yuan et al., 2014; Zou et al., 2014; Kong et al., 2015; Reddemann et al., 2015; Chang et al., 2016; Dong et al., 2016; Gulluoglu et al., 2016; Salem et al., 2016; Zhu et al., 2016), asthma (Jude et al., 2012; Dileepan et al., 2016), osteoarthritis (Rasheed et al., 2017), and rheumatoid arthritis (Peng J. S. et al., 2016). Studies on mouse and rat models have also proved its involvement in spermatogenesis (Luo et al., 2015) and testis differentiation (Rakoczy et al., 2013), chondrogenesis and growth (Pando et al., 2012; Waki et al., 2016), and sensitivity of fetal neural development to ethanol and nicotine (Balaraman et al., 2012). Circulating miR-140-3p has been identified as dysregulated in either plasma or serum from patients with different pathological conditions and it has already been suggested as a potential biomarker for some of them, like myotonic dystrophy type 1 and type 2 (Perfetti et al., 2014, 2016), biliary atresia (Peng X. et al., 2016), papillary thyroid carcinoma (Li et al., 2015), wet age-related macular degeneration (Ertekin et al., 2014), and myasthenia gravis (Nogales-Gadea et al., 2014). Its potential as non-invasive intracellular biomarker has also been demonstrated on blood samples from patients affected by coronary artery disease (Taurino et al., 2010; Karakas et al., 2017) and type 2 diabetes mellitus, (Collares et al., 2013). Finally, it has already been associated with two psychiatric mood disorders: it is upregulated in whole blood of bipolar disorder patients (Maffioletti et al., 2016) and major depression patients after 12 weeks of antidepressant treatment (Bocchio-Chiavetto et al., 2013).

We found a positive correlation and a linear relationship between ΔCt values for miR-140-3p and YGTSS scores of all study participants (Figure 2). Lower serum levels of miR-140-3p, which we observed in TS+ASD patients, could be potentially associated with both occurrence and worsening of motor and phonic tics, whereas higher serum levels of miR-140-3p, which we found in ASD patients, could be linked to the absence of tics. This finding indicates that expression analysis of serum miR-140-3p could strengthen the clinical diagnostic process of either ASD or TS+ASD. Moreover, this result gives strength to the hypothesis that the presence of phonic and motor tics, determining the comorbidity of TS with ASD, may be responsible for the different levels of serum miR-140-3p between ASD and TS+ASD patients.

Through network functional analysis, we observed that the regulatory network mediated by miR-140-3p is partly involved in managing structural and functional integrity of the nervous system and in the development of several human systems and organs (Table 3, Supplementary Figure 5). In particular, our computational data showed that CD38 and NRIP1, validated targets of miR-140-3p, take part in a set of biological processes convergingly dysregulated in ASD, like synaptic plasticity, immune response, and chromatin binding (Voineagu and Eapen, 2013; Gokoolparsadh et al., 2016; Ansel et al., 2017; Tables 4, 5, Supplementary Figures 6, 7).

CD38 was initially identified as an activation marker of immune cells, but it is now considered a virtually ubiquitous multifunctional molecule, involved in signaling and cell homeostasis. It is highly expressed in the brain, particularly in the hypothalamus (Quarona et al., 2013). Thanks to its ADP-ribosyl cyclase activity, CD38 regulates the mobilization of calcium ion from intracellular stores and therefore, it is mainly involved in proliferation, contraction, and secretion. It has also been found in a soluble form, maintaining this enzymatic activity, in body fluids and in exosomes (Quarona et al., 2013). CD38 has been linked to HIV infection, cancer, type 2 diabetes mellitus, and asthma (Quarona et al., 2013). Its enzymatic activity is responsible for the secretion of oxytocin and makes it one of the principal regulators of the social brain (Jin et al., 2007). Other than in social behavior, CD38 plays a role also in hippocampus-dependent learning and memory (Kim et al., 2016) and in postnatal glial development (Hattori et al., 2017). It is not surprising that much evidence has linked CD38 to ASD. Two genetic variants of CD38, the intronic SNP rs3796863 and the common Japanese SNP rs1800561 (causing the R140W mutation), have been associated with ASD (Munesue et al., 2010). In a study on two young sisters with ASD, a deletion of 4p15.32, resulting in a BST1 (*bone marrow stromal cell antigen 1*)—CD38 fusion transcript and in disruption of CD38 expression, was identified only in the girl affected by more severe ASD and asthma (Ceroni et al., 2014). CD38 expression is markedly reduced in LBC derived from ASD patients compared to their unaffected parents (Lerer et al., 2010); all-trans retinoic acid can upmodulate CD38 expression in these cells (Riebold et al., 2011). Finally, ASD patients have a higher absolute number of B cells per volume of blood and number of B cells expressing the cellular activation marker CD38 (Ashwood et al., 2011).

NRIP1 (also known as RIP140, *receptor-interacting protein 140*) is a widely expressed, multifaceted transcription co-regulator. Its primary physiological action is to trigger hormone-controlled gene suppression (Nautiyal et al., 2013). NRIP1 mainly controls female fertility in the ovary, promoting ovulation, and energy homeostasis in metabolic tissues. It exerts a co-activator function in the regulation of circadian rhythms, inflammatory cascade, and mammary gland development (Nautiyal et al., 2013). NRIP1 is also expressed in the cortical and hippocampal areas of the brain (Nautiyal et al., 2013). It plays a crucial role in brain development and functioning and in cognitive and emotional processes (Duclot et al., 2012; Flaisher-Grinberg et al., 2014; Feng et al., 2015). Downregulation of its nuclear form controls mice brain aging (Ghosh and Thakur, 2009) whereas its cytosolic form acts as a neuroprotector in mice brain, preventing endoplasmic reticulum stress-induced neuronal apoptosis (Feng et al., 2014) and maintaining brain cholesterol homeostasis (Feng et al., 2015). In mice hippocampus, increased levels of NRIP1 expression are associated with depression-like symptoms (Chunhua et al., 2016). NRIP1 protein levels are considerably increased in the hippocampus from Down Syndrome patients (Gardiner, 2006). NRIP1 has been suggested as a potential candidate gene in autism, on the basis of in silico analysis of chromosomal regions involved in an unbalanced rearrangement del(21)(q11.2q21.2), identified in an ASD patient from a cohort of 126 ASD patients through high-resolution comparative genome hybridization (Iurov et al., 2010). Its potential etiological role in ASD has not been further investigated, but evidence suggests that NRIP1 and ASD could be indirectly linked. NRIP1 represents a molecular bridge between circadian rhythms and metabolism. It is part of a feedback mechanism regulating the circadian clock: it is under circadian regulation and it can alter basal levels of other clock genes, also by acting as a coactivator for the nuclear receptor RORα, known to be a stimulator of clock genes' transcription (Poliandri et al., 2011). RORA has been identified as dysregulated in many tissues from ASD patients (Cook et al., 2015). In general, mutations affecting the function of circadian-relevant genes are more frequent in ASD patients than in unaffected controls (Yang et al., 2016). All these findings give strength to other results that have previously linked ASD and circadian clock genes, leading to the interpretation of ASD as a neurodevelopmental disorder arising from atypical biological and behavioral rhythms (Yang et al., 2016): NRIP1 contribution to this association is worthy of further investigation.

When studying circulating miRNAs in pathologies, the biggest challenge is to elucidate the relationship between the diseased tissue and the corresponding expression levels of these molecules observed in liquid biopsies. MiRNAs in circulation could either be passively released non-specific by-products of cellular activity and cell death or actively secreted cell-cell signaling messengers (Ragusa et al., 2014, 2015; Larrea et al., 2016). In ASD, this challenge is further complicated by the fact that a specific and unique diseased tissue has not been identified yet. In a gene expression dataset obtained from the analysis of whole blood samples of ASD patients, we found a marked dysregulation of twelve node genes from miR-140-3p-mediated network (Figure 3, Supplementary File 2). Even though we have detected the differential expression of some network node genes in all six ASD datasets reported, no one of those genes showed a marked and consistent expression trend in two or more datasets. Although it is not unexpected that independent microarray studies, using different technologies and platforms, give inconsistent results, we could not demonstrate a striking involvement of dysregulation of miR-140-3p-mediated network in ASD. Given the expression of miR-140-3p, CD38, and NRIP1 in the brain, it is also conceivable that brain tissues are responsible for serum dysregulation of miR-140-3p. Focusing on the contribution of serum extracellular vesicles to the expression of circulating miR-140-3p in ASD patients might help clarify potential tissue-serum links (Witwer, 2015). Nevertheless, our computational analysis of the potential functional role of intracellular miR-140-3p and of its possible involvement in ASD etiology suggests to the scientific community new processes, molecules and mechanisms to further investigate in the context of ASD.

Through ROC curve analyses and performance evaluation of predictive models, we proved that serum levels of miR-140-3p could be used in the discrimination of ASD patients from NCs, TS patients, and TS+ASD patients (Figures 4–6). We obtained the higher performance of serum miR-140-3p as a biomarker for the discrimination among ASD and TS+ASD patients. It was crucial to evaluate the performance of the biomarker through CV and permutation testing, since these predictive models were based on a miRNA already identified as differentially expressed between compared groups. We suggest that serum miR-140-3p could serve as a potential non-invasive biomarker to complement and support the behavior-based diagnosis of ASD, especially the differential one between ASD and TS+ASD. The main limit of our biomarker analysis is that we had only one miRNA to test for its predictive accuracy. It is well known that a single biomarker can hardly be as performing as a combination of them. Further studies on larger cohorts and on participants of lower age would be necessary in order to get compelling evidences on miR-140-3p discriminatory power and prove its value in supporting early diagnosis of ASD. In this context, it would be interesting to identify which factors can be responsible for ASD patient misclassification (Figures 6 1–3D) by miR-140-3p, in order to optimize its predictive performance.

Our study, just like many others on circulating miRNAs to be used as biomarkers, has some limitations. The first one regards diagnostic specificity. As reported above, circulating miR-140-3p has already been associated with multiple pathological conditions and this denote that it could be simply indicative of a general disease state (i.e., inflammation and response to stress). The second one is reproducibility. There is little overlap between circulating miRNAs reported as biomarkers from independent investigators and this challenges their clinical utility. Just one of two other independent studies on circulating miRNA in ASD (discussed below) is consistent with ours. That is why results should be validated in larger cohorts and experimental conditions should be carefully standardized (Witwer, 2015).

Our study is the third high-throughput one profiling circulating miRNAs in a body fluid from ASD patients in order to discover some potential biomarkers.

The first study (Mundalil Vasu et al., 2014) was carried out on serum from a Japanese cohort of 55 ASD patients and 55 unaffected controls. The authors identified and validated 13 circulating miRNAs as dysregulated in serum from ASD patients and showed the accurate predictive power of 5 of them in discriminating ASD patients (Mundalil Vasu et al., 2014). None of circulating miRNAs from this study matches those from our profile. Our ASD and NC sample size is smaller than the one from this work, but we have used an array technology that allowed us to profile the expression of many more miRNAs than the 139 that the authors analyzed.

We tested the expression of these 5 predictive miRNAs (miR-19b-3p, miR-130a-3p, miR-181b-5p, miR-320a, miR-572) (Mundalil Vasu et al., 2014), together with miR-429 from our previous investigation on TS (Rizzo et al., 2015), in sera from 15 ASD patients, 15 TS patients, and 15 NCs. We did not observe any difference in miRNA expression among groups with the exception of miR-429, which we confirmed as DE between TS patients and NCs. Differences in sample size (55 samples per group vs. 15 samples per group) did not alter the result on miR-429 expression (Rizzo et al., 2015). However, functional enrichment analyses from both studies (Mundalil Vasu et al., 2014; this paper) demonstrated over-representation of the same neurological pathways, as TGF-β signaling, Hedgehog signaling, Wnt signaling, and regulation of synaptic plasticity. This observation suggests that discrepancies can be explained with differences in pre-analytic variables, such as genetic structure of studied populations, cohort composition, sample processing, validation technique, and data normalization (Witwer, 2015). Ethnicity of participants, cohort size, and miRNA panel and intercalating dye-based system used may have determined the inconsistencies observed.

The second paper (Hicks et al., 2016) describes a pilot study on saliva from a US cohort of 24 ASD patients and 21 unaffected controls, whose results partly match with ours. By RNA-sequencing, the authors identified 14 circulating miRNAs as dysregulated in saliva from ASD patients and showed the discriminative accuracy of this molecular signature (Hicks et al., 2016). MiR-140-3p is part of this ASD molecular fingerprint and is upregulated in ASD patients compared to unaffected controls, as in our study (Hicks et al., 2016; this paper). Moreover, in agreement with our results, their functional enrichment analysis detected significant over-representation of target genes related to neuronal development and transcriptional activation (Hicks et al., 2016). Our ASD and NC sample size is slightly bigger than the one from this work. Also, sequencing data from it have not been validated through miRNA-specific qPCR assays. Discrepancies between this work and our study can also be explained, other than with all the factors listed above, with the fact that they investigated two different human body fluids. It is interesting that miR-140-3p shows the same expression trend in both saliva and serum. This observation may suggest that shared mechanisms could determine the increased levels of miR-140-3p in both body fluids.

These two studies, differently from ours, have identified more than one biomarker for ASD. Furthermore, they have focused only on ASD patients and NCs, whereas our study is the first high-throughput one profiling circulating miRNAs also in patients suffering from another neurodevelopmental disorder comorbid with ASD, TS+ASD patients.

Through the identification of a serum biomarker, our study provides insight into concealed molecular mechanisms determining ASD and a potential complementary and supportive mean for a simpler, faster, and unbiased ASD diagnosis. The network that miR-140-3p regulates is involved in a set of biological processes convergingly dysregulated in ASD. Molecular characterization of miR-140-3p network would contribute to further clarify the heterogeneous molecular basis of ASD. Moreover, serum miR-140-3p could potentially be used as a non-invasive biomarker for ASD, easy to test through liquid biopsies.

## Ethics statement

This study was carried out in accordance with the recommendations of the local Ethics Committee (Comitato Etico dell' Università degli Studi di Catania, CEU) with written informed consent from all subjects.

## Author contributions

MP and RR conceived the project with contributions by CDP, MR, DB, and MC. MP, CDP, MR, DB, and MC designed the experiments. MC and CB performed them. RR, MG, CND, and RB carried out patients' recruitment and clinical data analysis. RR performed clinical diagnosis. MC performed computational analysis and carried out statistical data analysis. MP, RR, and MC wrote the paper. All authors contributed to the critical revision of the data, read and approved the final manuscript.

### Conflict of interest statement

The authors declare that the research was conducted in the absence of any commercial or financial relationships that could be construed as a potential conflict of interest.

## Footnotes

^1^http://mev.tm4.org

^2^www.graphpad.com

^3^http://diana.imis.athena-innovation.gr/DianaTools/index.php?r=tarbase/index

^4^http://mirtarbase.mbc.nctu.edu.tw/

^5^www.R-project.org/

^6^www.ncbi.nlm.nih.gov/geo/info/geo2r.html

^7^http://http://www.metaboanalyst.ca/

## Supplementary material

Supplementary Figure 1 MiR-140-3p-mediated regulatory network. Nodes representing MIR140 gene and direct target genes of miR-140-3p are depicted as red diamonds; those symbolizing protein-coding genes are drawn as yellow circles; those standing for miRNA-coding genes are illustrated as orange triangles. Size of both nodes and node labels, except for CD38, MIR140, and NRIP1, is directly proportional to node degree within the network

Supplementary Figure 2 Over-represented GO terms in miR-140-3p-mediated regulatory network compared to the entire genome. **(A)** Dot plot for GO Biological Process terms. **(B)** Dot plot for GO Molecular Function terms. **(C)** Dot plot for GO Cellular Component terms. Each circle in the plots symbolizes an over-represented term: its x-axis coordinate reflects the Gene Ratio value; its size is directly proportional to the Count value; its color represents the Benjamini-Hochberg adjusted *p*-value generated by the hypergeometric test. Gene Ratio: the ratio of number of genes of interest that are annotated with a certain term from the database used to perform the analysis to number of genes of interest that are annotated with terms from the same database. Count: number of node genes within the network that are annotated with a certain term. GO: Gene Ontology

Supplementary Figure 3 Over-represented KEGG and Reactome terms in miR-140-3p-mediated regulatory network compared to the entire genome. **(A)** Dot plot for KEGG Pathway terms. **(B)** Dot plot for Reactome Pathway terms. For an in-depth description of the plots see Supplementary Figure 2 legend. KEGG: Kyoto Encyclopedia of Genes and Genomes.

Supplementary Figure 4 Over-represented DO terms in miR-140-3p-mediated regulatory network compared to the entire genome. Dot plot for DO terms. For an in-depth description of the plot see Supplementary Figure 2 legend. DO: Disease Ontology.

Supplementary Figure 5 Over-represented GO Biological Process terms regarding nervous system and development in miR-140-3p-mediated regulatory network. Dot plot for all the most interesting GO Biological Process terms regarding nervous system and development. Each symbol in the plot symbolizes an over-represented term: its x-axis coordinate reflects the Gene Ratio value; its y-axis coordinate reflects the Background Ratio value; its color represents the Benjamini-Hochberg adjusted *p*-value generated by the hypergeometric test. Gene Ratio: the ratio of number of genes of interest that are annotated with a certain term from the database used to perform the analysis to number of genes of interest that are annotated with terms from the same database. Background Ratio: the ratio of number of genes in the genome that are annotated with a certain term from the database used to perform the analysis to number of genome genes that are annotated with terms from the same database. GO: Gene Ontology.

Supplementary Figure 6 Over-represented GO, DO and KEGG terms associated with CD38 in miR-140-3p-mediated regulatory network. Dot plot for all the most interesting GO, DO and KEGG terms whose enrichment is determined by CD38. For an in-depth description of the plot see Supplementary Figure 5 legend. For more information about what symbol shapes stand for see figure legend. GO terms whose enrichment is determined by both CD38 and NRIP1 are represented by bigger symbols. GO: Gene Ontology; DO: Disease Ontology; KEGG: Kyoto Encyclopedia of Genes and Genomes.

Supplementary Figure 7 Over-represented GO and Reactome terms associated with NRIP1 in miR-140-3p-mediated regulatory network. Dot plot for all the most interesting GO and Reactome terms whose enrichment is determined by NRIP1. For an in-depth description of the plot see Supplementary Figure 5 legend. For more information about what symbol shape stands for see figure legend. GO terms whose enrichment is determined by both CD38 and NRIP1 are represented by bigger symbols. GO: Gene Ontology.

Supplementary File 1 MiRNA profiling raw data: Ct values for TLDA panel A and B. Customized normalization approach.

Supplementary File 2 Data from network expression analysis.

Supplementary File 3 ΔCt values for miR-30d, miR-140-3p, miR-148a^*^, and miR-222.

Supplementary File 4 ΔCt values for miR-140-3p and neuropsychiatric scores of all participants.

Supplementary File 5 Data from network functional analysis.

## Abbreviations

ADHD: attention deficit/hyperactivity disorder
ADI-R: Autism Diagnostic Interview-Revised
ADOS: Autism Diagnostic Observation Schedule
ASD: Autism spectrum disorder
AUC: Area under the ROC curve
BH: Benjamini-Hochberg
BST1: bone marrow stromal cell antigen 1
CCDC85B: coiled-coil domain containing 85B
CD38: CD38 molecule
CD3E: CD3e molecule
CI: Confidence Interval
CIB1: calcium and integrin binding 1
CTBP1: C-terminal binding protein 1
CV: cross-validation
DE: differentially expressed
DO: Disease Ontology
DSM-5: Diagnostic and Statistical Manual of Mental Disorders, 5th Edition
DSM-IV-TR: Diagnostic and Statistical Manual of Mental Disorders, IV edition–Text Revision
E2F1: E2F transcription factor 1
EP300: E1A binding protein p300
ESR1: estrogen receptor 1
F: female
FC: Fold Change
FDR: False Discovery Rate
GMN: global median normalization
GO: Gene Ontology
IQ: Intelligence Quotient
KEGG: Kyoto Encyclopedia of Genes and Genomes
LBC: lymphoblastoid cell lines
LCK: LCK proto-oncogene, Src family tyrosine kinase
M: male
MAP3K7: mitogen-activated protein kinase kinase kinase 7
MIR140: microRNA 140
MiRNA: MicroRNA
mTOR: mammalian target of rapamycin
N: no
NC: unaffected control
NCOR: nuclear receptor co-repressor
NR1H2: nuclear receptor subfamily 1 group H member 2
NRIP1: nuclear receptor interacting protein 1
NT: Not Tested
OCD: obsessive-compulsive disorder
ODD: oppositional defiant disorder
PHF8: PHD finger protein 8
PI3K: phosphatidylinositol-4,5-bisphosphate 3-kinase
POLR2A: RNA polymerase II subunit A
RARA: retinoic acid receptor alpha
RIP140: receptor-interacting protein 140
ROC: Receiver Operator Characteristic
RORA: RAR related orphan receptor A
SAM: Significance of Microarrays Analysis
SD: standard deviation
STAT3: signal transducer and activator of transcription 3
SWI/SNF: switch/sucrose non-fermentable
TAF1: TATA-box binding protein associated factor 1
TLDA: TaqMan Low Density Array
TS: Tourette Syndrome
WISC-III: Wechsler Intelligence Scale for Children, III edition
Y: yes
YGTSS: Yale Global Tic Severity Scale
ZAP70: zeta chain of T cell receptor associated protein kinase 70.
