# Supplementary figures and images for: Expression and Regulatory Network Analysis of miR-140-3p, a New Potential Serum Biomarker for Autism Spectrum Disorder
Source: Front Mol Neurosci. 2017 Aug 10;10:250. doi: 10.3389/fnmol.2017.00250 (PMC5554380; doi:10.3389/fnmol.2017.00250)

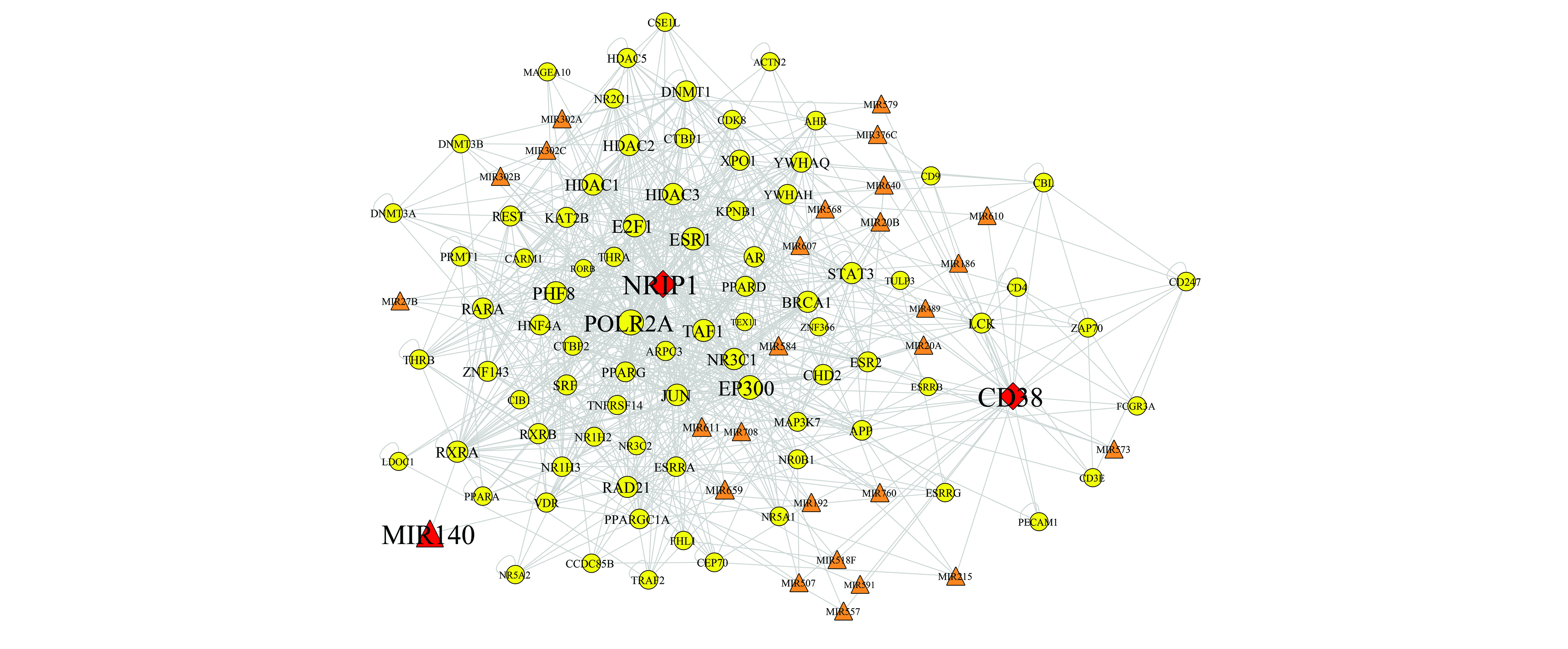

Supplement: Supplementary Figure 1 — MiR-140-3p-mediated regulatory network. Nodes representing MIR140 gene and direct target genes of miR-140-3p are depicted as red diamonds; those symbolizing protein-coding genes are drawn as yellow circles; those standing for miRNA-coding genes are illustrated as orange triangles. Size of both nodes and node labels, except for CD38, MIR140, and NRIP1, is directly proportional to node degree within the network [file Image1.JPEG]

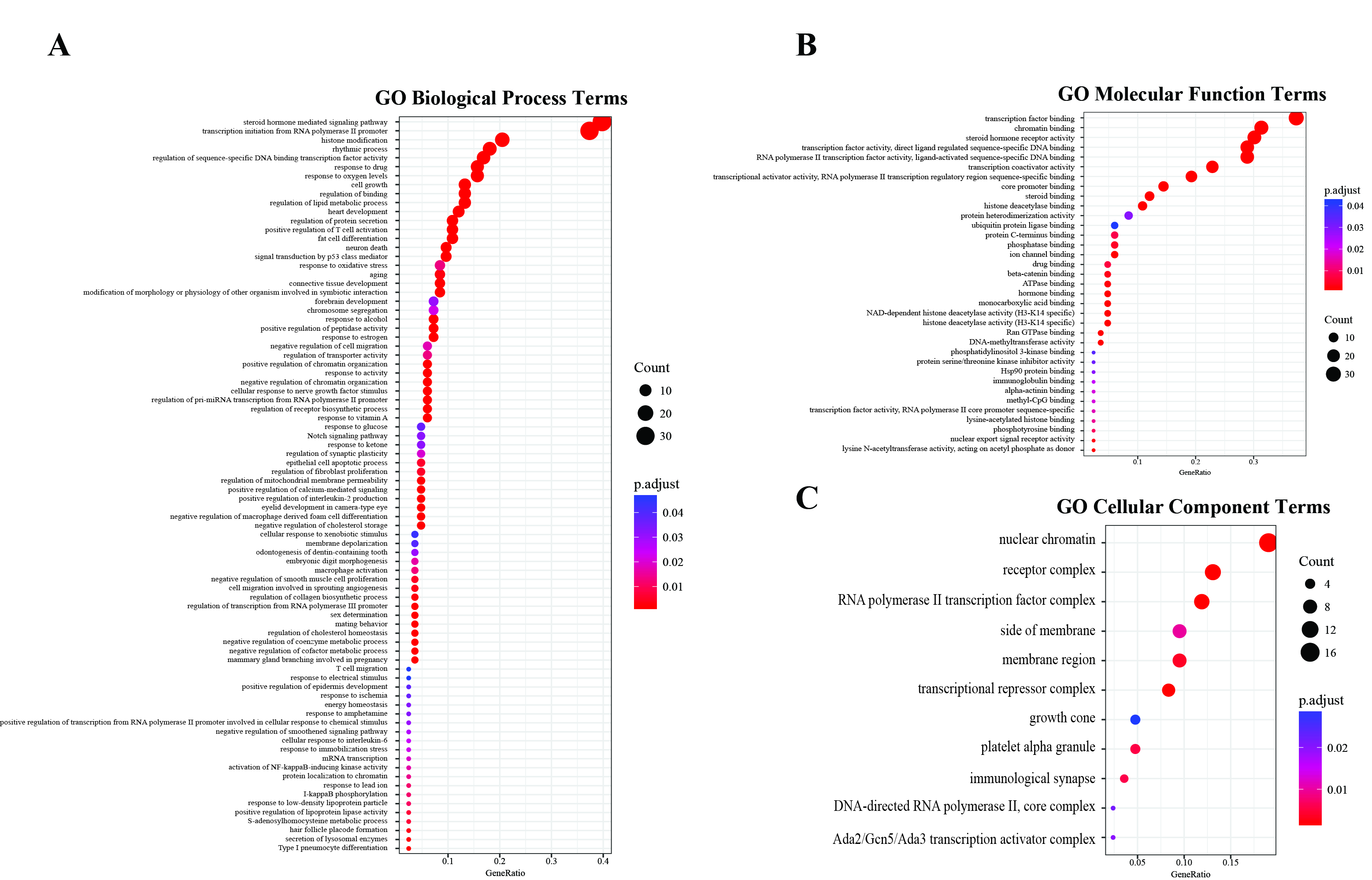

Supplement: Supplementary Figure 2 — Over-represented GO terms in miR-140-3p-mediated regulatory network compared to the entire genome. (A) Dot plot for GO Biological Process terms. (B) Dot plot for GO Molecular Function terms. (C) Dot plot for GO Cellular Component terms. Each circle in the plots symbolizes an over-represented term: its x-axis coordinate reflects the Gene Ratio value; its size is directly proportional to the Count value; its color represents the Benjamini-Hochberg adjusted p-value generated by the hypergeometric test. Gene Ratio: the ratio of number of genes of interest that are annotated with a certain term from the database used to perform the analysis to number of genes of interest that are annotated with terms from the same database. Count: number of node genes within the network that are annotated with a certain term. GO: Gene Ontology [file Image2.JPEG]

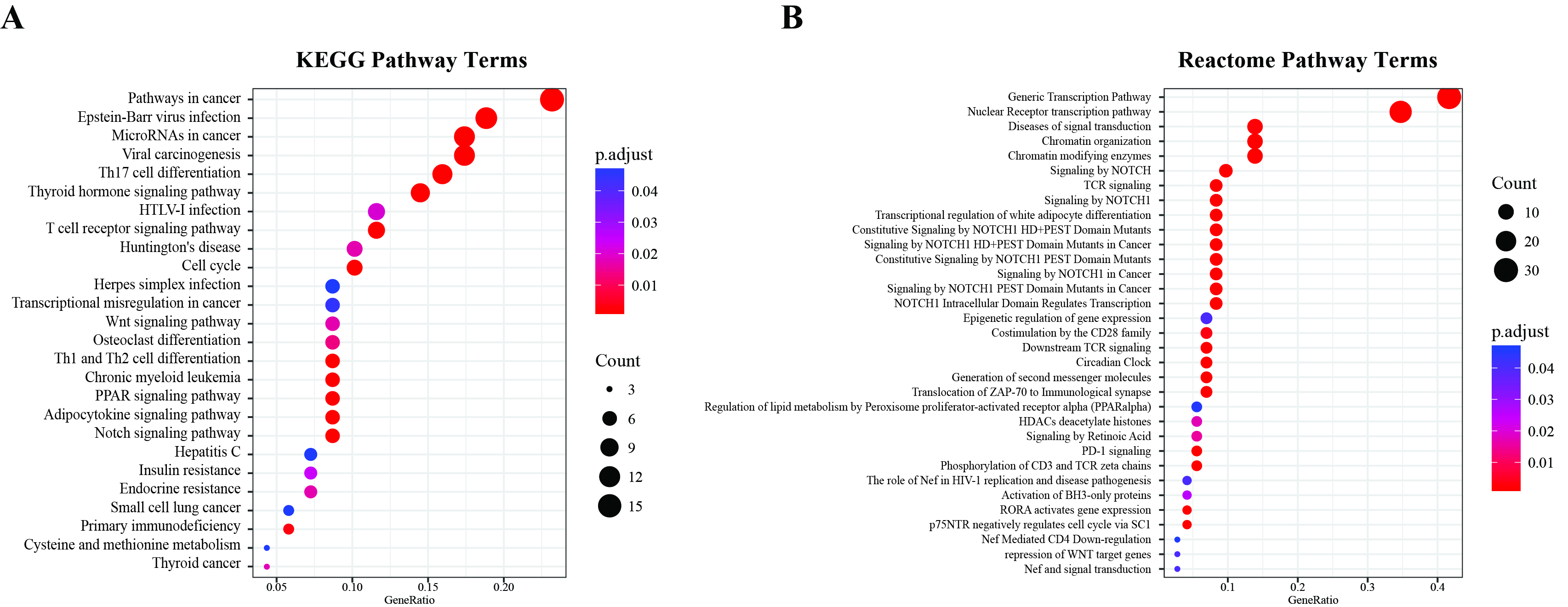

Supplement: Supplementary Figure 3 — Over-represented KEGG and Reactome terms in miR-140-3p-mediated regulatory network compared to the entire genome. (A) Dot plot for KEGG Pathway terms. (B) Dot plot for Reactome Pathway terms. For an in-depth description of the plots see Supplementary Figure 2 legend. KEGG: Kyoto Encyclopedia of Genes and Genomes. [file Image3.JPEG]

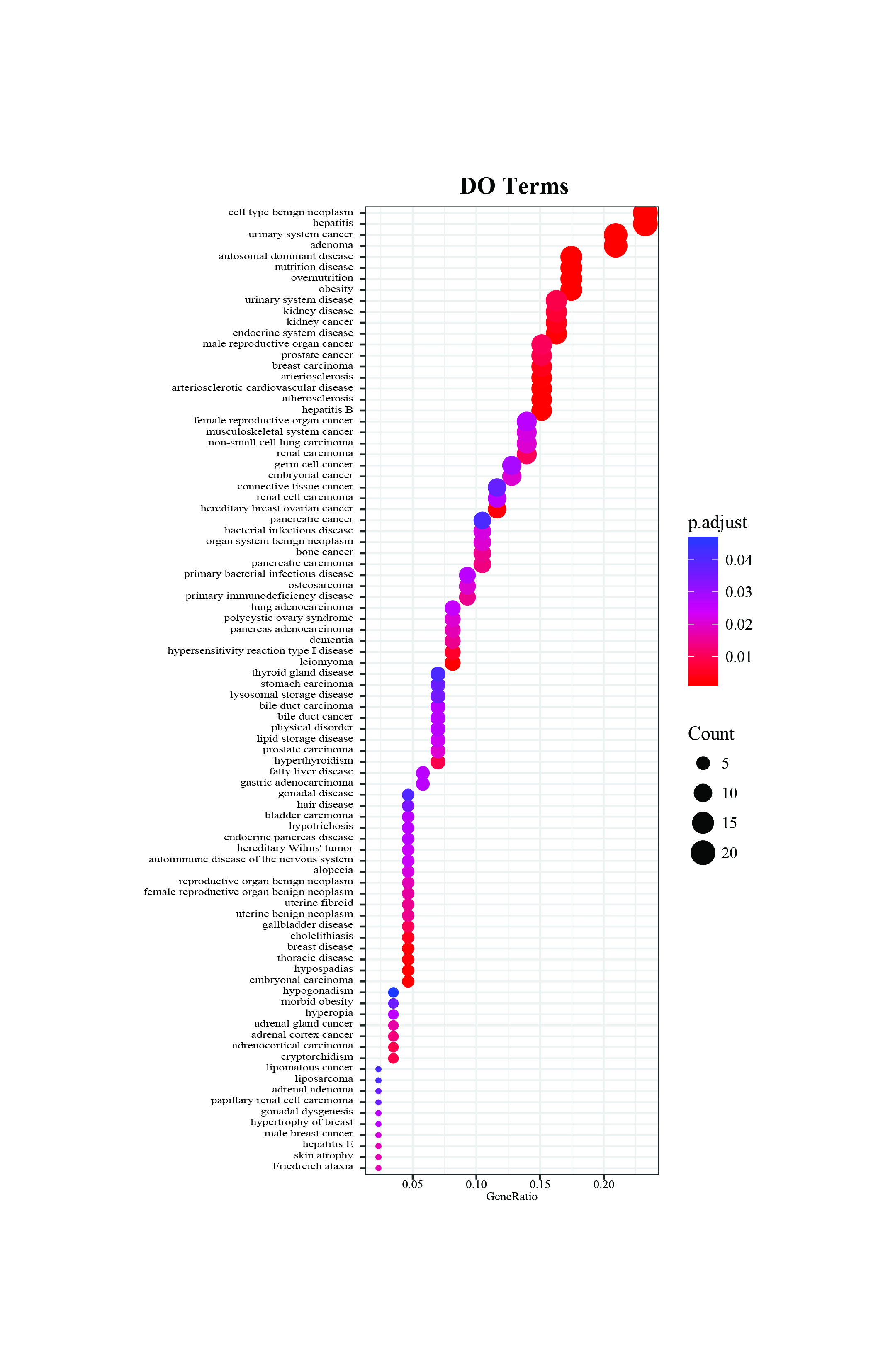

Supplement: Supplementary Figure 4 — Over-represented DO terms in miR-140-3p-mediated regulatory network compared to the entire genome. Dot plot for DO terms. For an in-depth description of the plot see Supplementary Figure 2 legend. DO: Disease Ontology. [file Image4.JPEG]

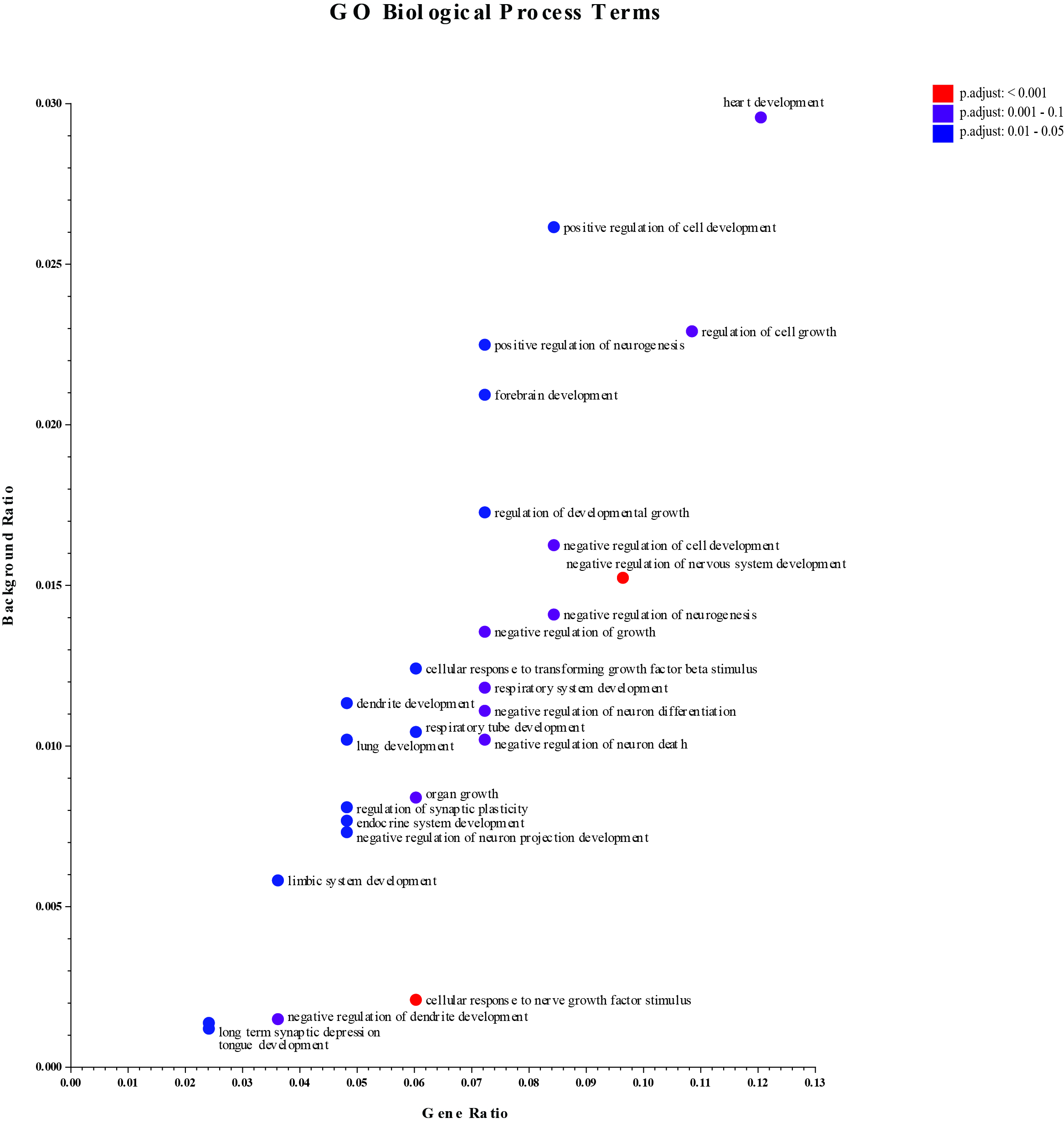

Supplement: Supplementary Figure 5 — Over-represented GO Biological Process terms regarding nervous system and development in miR-140-3p-mediated regulatory network. Dot plot for all the most interesting GO Biological Process terms regarding nervous system and development. Each symbol in the plot symbolizes an over-represented term: its x-axis coordinate reflects the Gene Ratio value; its y-axis coordinate reflects the Background Ratio value; its color represents the Benjamini-Hochberg adjusted p-value generated by the hypergeometric test. Gene Ratio: the ratio of number of genes of interest that are annotated with a certain term from the database used to perform the analysis to number of genes of interest that are annotated with terms from the same database. Background Ratio: the ratio of number of genes in the genome that are annotated with a certain term from the database used to perform the analysis to number of genome genes that are annotated with terms from the same database. GO: Gene Ontology. [file Image5.JPEG]

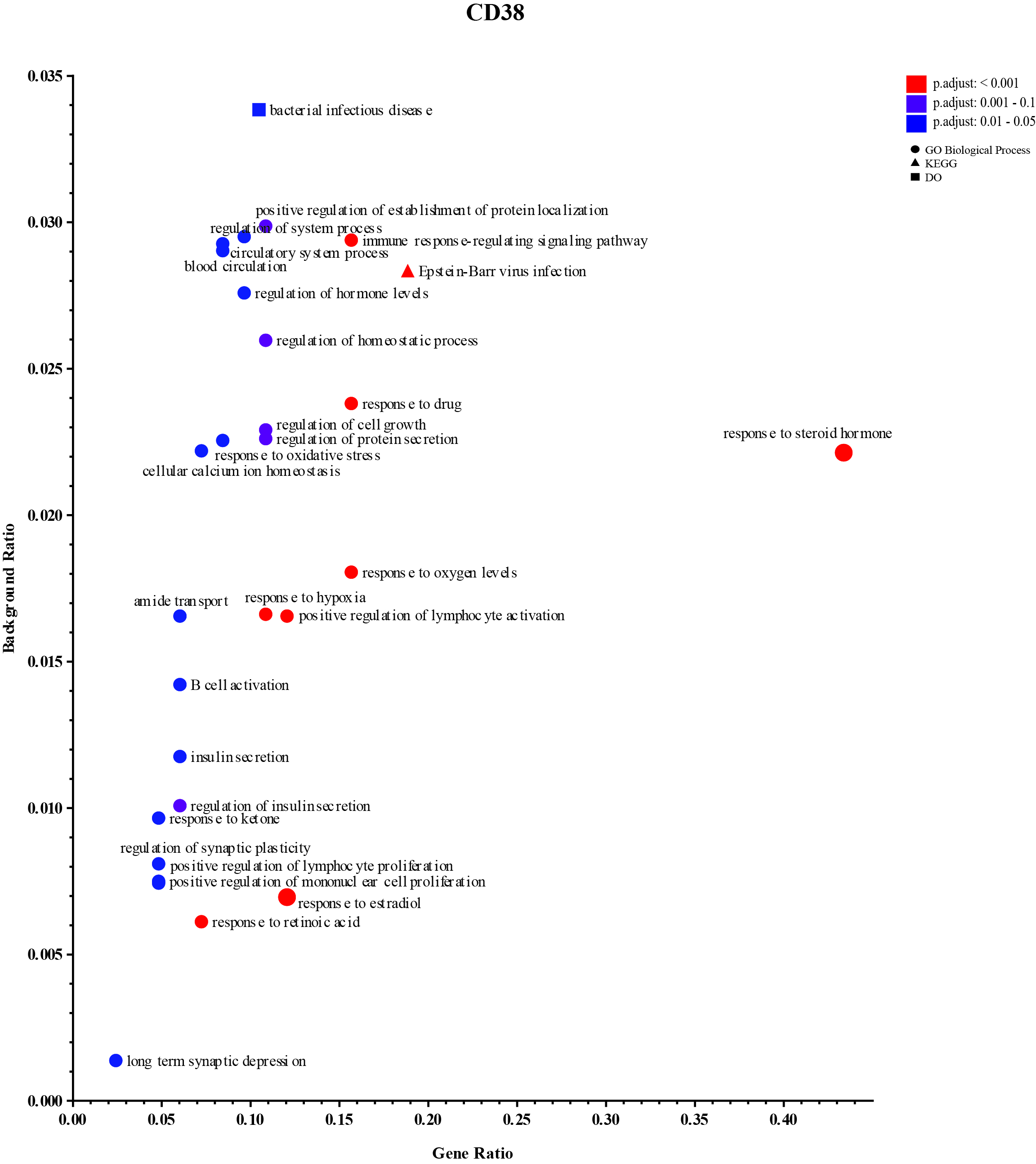

Supplement: Supplementary Figure 6 — Over-represented GO, DO and KEGG terms associated with CD38 in miR-140-3p-mediated regulatory network. Dot plot for all the most interesting GO, DO and KEGG terms whose enrichment is determined by CD38. For an in-depth description of the plot see Supplementary Figure 5 legend. For more information about what symbol shapes stand for see figure legend. GO terms whose enrichment is determined by both CD38 and NRIP1 are represented by bigger symbols. GO: Gene Ontology; DO: Disease Ontology; KEGG: Kyoto Encyclopedia of Genes and Genomes. [file Image6.JPEG]

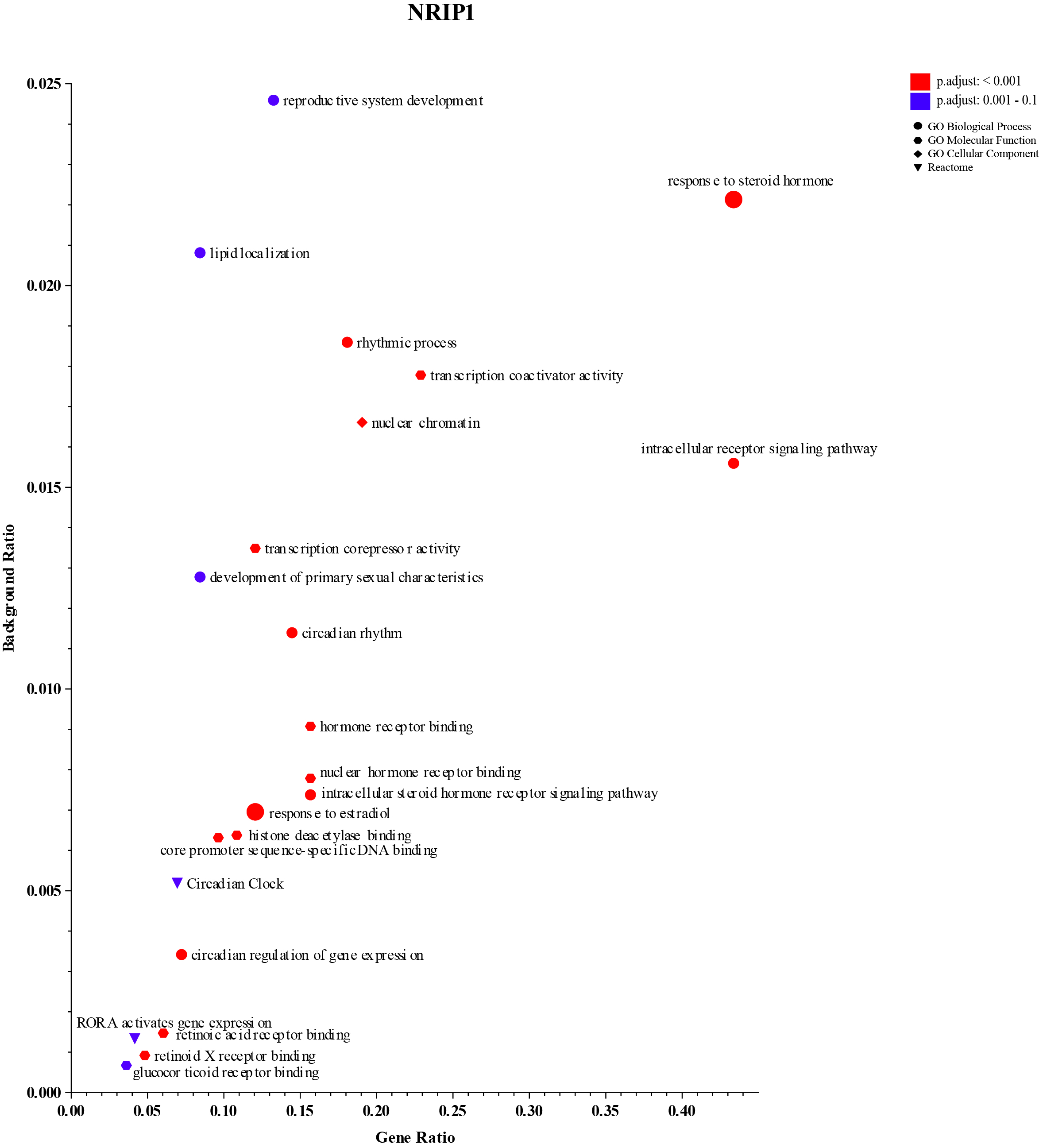

Supplement: Supplementary Figure 7 — Over-represented GO and Reactome terms associated with NRIP1 in miR-140-3p-mediated regulatory network. Dot plot for all the most interesting GO and Reactome terms whose enrichment is determined by NRIP1. For an in-depth description of the plot see Supplementary Figure 5 legend. For more information about what symbol shape stands for see figure legend. GO terms whose enrichment is determined by both CD38 and NRIP1 are represented by bigger symbols. GO: Gene Ontology. [file Image7.JPEG]
